# Shape of the hemodynamic response function in deaf infants eligible for cochlear implantation

**DOI:** 10.1117/1.NPh.13.S1.S13012

**Published:** 2026-03-31

**Authors:** Judit Gervain, Gaia Lucarini, Davide Brotto, Valeria Del Vecchio, Benedetta Colavolpe, Ramon Guevara, Caroline Nallet, Nicole Galoforo, Elisa Lovato, Giusy Melcarne, Alessandro Martini, Domenico d’Errico, Salvatore Allosso, Elena Cantone, Gino Marioni, Andrea De Bartolomeis, Patrizia Trevisi, Anna Rita Fetoni

**Affiliations:** a University of Padua, Department of Developmental and Social Psychology, Padua, Italy; b University of Padua, Padova Neuroscience Center, Padua, Italy; c Université Paris Cité & CNRS, Integrative Neuroscience and Cognition Center, Paris, France; d University of Padua, Section of Otolaryngology, Department of Neuroscience DNS, Padua, Italy; e Federico II University Hospital, Department of Head and Neck, Hearing and Balance Unit, Naples, Italy; f University of Padua, Department of Physics and Astronomy, Padua, Italy; g Federico II University, Department of Neuroscience, Reproductive Science and Dentistry, Unit of Otolaryngology, Naples, Italy; h University of Calabria, University of Calabria Hospital, Unit of Otolaryngology, Cosenza, Italy; i University of Padua, Department of Neuroscience DNS, Phoniatrics and Audiology Unit, Treviso, Italy; j Federico II University, Department of Neuroscience, Reproductive Science and Dentistry, Unit of Psychiatry, Naples, Italy; k Federico II University, Department of Neuroscience, Reproductive Science and Dentistry, Unit of Audiology, Naples, Italy

**Keywords:** hemodynamic response function parameters, model fitting, infants, deafness, hearing loss, native language

## Abstract

**Significance:**

Deaf and hard-of-hearing infants’ hemodynamic response function (HRF) has not yet been characterized. However, without an appropriate estimate of their HRF, neuroimaging modalities relying on hemodynamic responses, e.g., functional magnetic resonance imaging (fMRI) or functional near-infrared spectroscopy (fNIRS), cannot be used reliably in this population, e.g., prior to and following cochlear implantation. We contribute to better theoretical models of and more suitable therapeutic interventions for the neural changes induced by auditory and language deprivation in deaf infants.

**Aim:**

We aim to characterize the parameters of the HRF of deaf and hard-of-hearing infants in response to Italian.

**Approach:**

We measured 2- to 20-month-old Italian-exposed infants’ HRF to Italian using fNIRS in the bilateral temporal, i.e., auditory, cortices. We characterized the HRF for all infants and for three clinically relevant subsets: (i) monolingual Italian infants, (ii) genetically deaf infants, and (iii) infants aged 5 to 12 months. We computed the following parameters: peak amplitude, time-to-peak, full width at half maximum, and where present, the amplitude and latency of the initial dip and/or the final undershoot, using a model-based parameter fitting approach. We statistically compared these HRF parameters to those of typically hearing infants.

**Results:**

Deaf and typically hearing infants showed largely similar HRFs, with both groups reaching comparable main peak amplitudes. Minor differences have been found in the latencies of some response components.

**Conclusions:**

Our results provide the first detailed characterization of the hemodynamic response to native-language speech in deaf and hard-of-hearing infants, improving clinical and therapeutic approaches through more accurate analysis of fMRI and fNIRS data.

## 1 Introduction

Several brain imaging techniques such as functional magnetic resonance imaging (fMRI) or functional near-infrared spectroscopy (fNIRS) measure the hemodynamic response, i.e., focal changes in the oxygenation of brain areas or circuits in response to stimulation or at rest, to understand perceptual, cognitive, affective, or other processing mechanisms in the brain. These neuroimaging techniques have allowed us to visualize the anatomy and the functioning of the human brain quickly, efficiently, and noninvasively, revolutionizing our understanding of how the brain gives rise to our perceptions, thoughts, feelings, beliefs, and desires.

For the accurate use of these neuroimaging techniques, a precise understanding of the hemodynamic response function (HRF) has been necessary. Indeed, the HRF is now well characterized in typical adults under standard experimental conditions.^1^^–^^4^ However, how the HRF develops early in life is much less well understood; as the infant brain is anatomically very immature, fetal hemoglobin has slightly different properties than regular hemoglobin, and the neuro-vascular coupling is not fully developed early in life.^5^^–^^9^ This knowledge gap is even greater in atypically developing infants such as preterm infants and infants born with genetic or other developmental disorders. However, a detailed characterization of the HRF in atypically developing infants would be particularly important both theoretically and clinically. Theoretically, it would enable better mechanistic models of developmental disorders, shedding light on the developmental processes of the brain. Clinically, more accurate HRFs would yield more precise diagnostic and therapeutic assessments, which are currently often based on HRFs derived from typically developing infants or even adults, leading to less precise estimates of the underlying neural mechanisms.

The current paper, therefore, aims to fill this knowledge gap by providing a detailed characterization of the HRF in deaf infants eligible for cochlear implantation. Describing the HRF in this population is of particular relevance for at least two reasons. First, deaf and cochlear-implanted infants’ brains may function differently from those of typically hearing infants due to factors such as the underlying causes of deafness, auditory deprivation, or a combination of the two. Cochlear implantation and the restoration of hearing trigger further plastic changes in the brain, and in most cases, oral language starts to emerge soon after implantation.^10^^–^^14^ It is of utmost clinical significance to explore and monitor these neural re-organization processes as their understanding can contribute to better therapeutic choices and enhanced implantation outcomes. However, as these infants’ brain development may follow an atypical trajectory,^15^^,^^16^ their HRFs and the underlying neuro-vascular coupling may also differ from those of typical infants. Second, in this population, fNIRS is particularly well suited for brain imaging as alternative methods are less feasible:^17^^,^^18^ fMRI is uncomfortable and difficult to perform with developmental populations such as deaf and cochlear-implanted children, whereas electroencephalography (EEG) is problematic due to signal contamination from the implant. fNIRS, by contrast, is readily compatible with most cochlear implants. It is also user-friendly and well tolerated even by the youngest infants.^9^ However, for accurate fNIRS measures, the underlying HRF needs to be characterized appropriately.

Existing fNIRS studies with cochlear-implanted infants and children have explored the neural mechanisms underlying auditory and language processing prior to and after implantation, shedding light on some of the plastic changes that occur as a result of deafness and the restoration of hearing.^17^^,^^19^^–^^30^ These findings of these studies have been summarized in a recent review.^31^ Importantly, however, these studies did not investigate the HRF itself in this population.

**Fig. 1:**
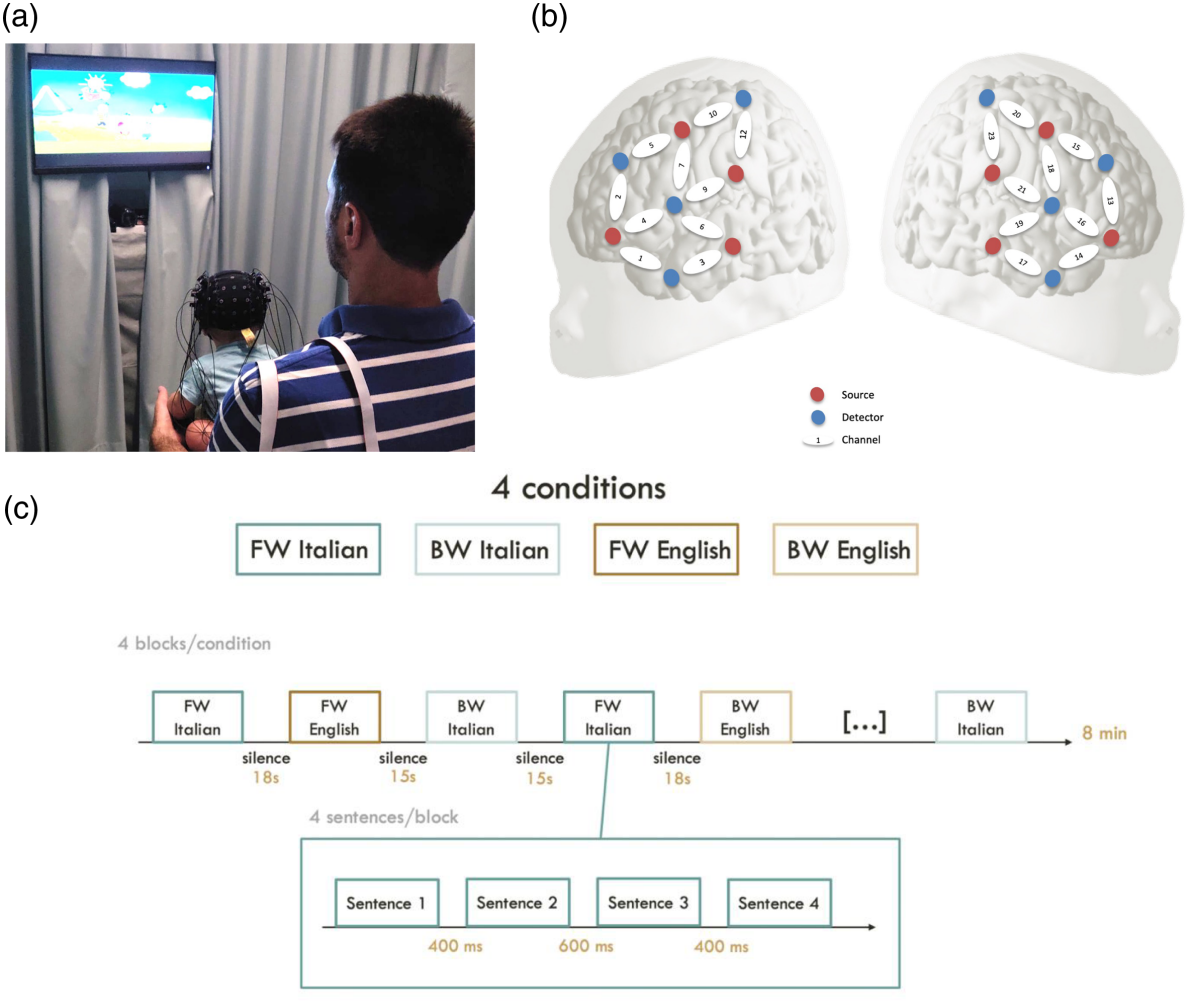
(a) Infant participant wearing the NIRS headgear in the experimental setup. (b) Optode configuration used in the study overlaid on a schematic infant brain. (c) The experimental design of the study.

To do so, the current study has tested the hemodynamic responses of deaf infants aged 2 to 20 months of age in the bilateral temporal areas, i.e., the auditory cortices^32^^–^^37^ [Figs. 1(a) and 1(b)]. We tested them using a standard language discrimination paradigm commonly used with typically hearing infants to assess speech perception and language acquisition skills,^35^^,^^38^^–^^42^ as this paradigm elicits robust hemodynamic responses in typical newborn infants. The paradigm consists of four conditions, presented using a simple block design. The conditions include (i) the native language of the majority of the infants in the sample, Italian (forward Italian, FWIT), (ii) the same stimuli played backward (backward Italian, BWIT), (iii) an unfamiliar and rhythmically different language, English (forward English, FWEN), and (iv) the nonnative stimuli played backward (backward English, BWEN). Using this paradigm, typically hearing newborns discriminate between their native language and rhythmically different languages,^38^^,^^40^^,^^43^^,^^44^ as well as between backward and forward speech in the native language,^35^^,^^38^ showing an increased HRF for the forward native language over the other three conditions. We have chosen to use this paradigm rather than a simpler, more basic auditory task, e.g., impulse-like beeps or pure tones, for multiple reasons. First, this task has been amply used in typical development and is thus known to elicit robust responses.^35^^,^^38^^–^^42^ Second, its simple block design is the most commonly used paradigm in developmental fNIRS studies. Describing the HRF triggered by this design is thus highly relevant for the developmental fNIRS community. Third, infants’ responses to the naturally spoken, ecologically valid stimuli of the forward native language condition are particularly relevant for understanding the neural mechanisms of speech perception, language development, hearing loss, and cochlear implantation outcomes. Nonlinguistic stimuli such as beeps or pure tones do not match or scale up to the complexity of speech and are therefore less suitable in this population.

The infants tested in this sample are candidates for cochlear implantation and thus have moderate, severe, or profound hearing loss. Importantly, however, as previous research indicates,^28^ their residual hearing, especially when supported by a hearing aid, still affords basic auditory discrimination abilities, e.g., discriminating the human voice from other sounds.^28^ The mechanisms behind these abilities, whether supported only by the residual hearing or possibly also through other modalities, e.g., vibrations/tactile sensations and visual experience with talking faces, are currently unknown. Indeed, these abilities typically remain uncharted and are not taken into account during the cochlear implantation (CI) training and rehabilitation process. However, they could serve as a basis for CI training and may be built on during therapy and language development after implantation. This requires a good understanding of the residual abilities.

Accordingly, we derive and characterize the HRF for the forward Italian condition, i.e., the language to which all infants in the sample are exposed and which serves as input for language acquisition. We characterize the HRF for all deaf infants, as well as for three clinically relevant subsets of our full deaf sample: (i) monolingual Italian infants, (ii) genetically deaf infants, who constitute the most frequent etiology in our sample, (iii) infants between 5 and 12 months of age, the most common age for implantation, accounting for the majority of our sample. We compute the following commonly used parameters to describe the HRFs:^1^^,^^2^^,^^4^ peak amplitude, time-to-peak (TTP), full width at half maximum (FWHM), and where present, the amplitude and latency of the initial dip and/or the final undershoot, using a model-based, parameter fitting approach. Finally, we statistically compare these parameters to those of typically hearing infants.

The comparison of the four conditions and other potential analyses is outside the scope of the current study and will be undertaken in subsequent work. The aim of the current study is to characterize the shape of the HRF in deaf and hard-of-hearing infants.

## 2 Materials and Methods

### 2.1 Participants

Forty-eight infants treated at the Audiology Service of the Otolaryngology Ward of the University Hospital of Padua or the Hearing and Balance Unit of the University Hospital of Naples were tested (mean age at the first testing session from which data are reported here: 386 days, age range: 66 to 1000 days, 21 females). Of these, eight infants did not attend this first test session and seven provided poor data (see Sec. 2.4 for details). Data from the remaining 33 infants (mean age: 314 days; age range: 66 to 618 days, 15 females) were entered into the final analysis. The demographic information, etiology of hearing impairment, and hearing thresholds are shown in Table 1. All infants were exposed to Italian at least 20% of the time. Some infants were also exposed to a second language, which was a spoken language for seven infants and Italian Sign Language for one infant (Table 1). Infants were tested with their hearing aids if they used them regularly, or without, if they typically did not use them in their everyday life, as our aim was to test them under the conditions that best characterized their daily hearing experience to increase ecological validity.

**Table 1:** Demographic information of the deaf participants who entered the current analysis.

Demographic characteristics							Auditory threshold with hearing aid	
#	Sex	Gestational age (week, days)	Birth weight (grams)	Age at test (days)	% Exposure to Italian (other language)	Possible etiology	Right (dB)	Left (dB)
1	m	38 w 5 d	3420	618	100	Genetic	87	101
2	m	40 w 2 d	3600	378	100	Genetic	118	118
3	f	40 w 2 d	3500	538	100	Neuropathy	>100	>100
4	m	40 w 0 d	NA	66	30 (Turkish)	Genetic	100	100
5	m	34 w 6 d	2670	314	100	Unknown	79	79
6	m	39 w 2 d	3290	342	100	Unknown	60	60
7	f	40 w 0 d	2880	609	100	Unknown	>90	>90
8	f	41 w 0 d	3940	315	100	Genetic	49	49
9	f	40 w 5 d	3560	482	100	Genetic	55	55
10	m	39 w 0 d	3500	352	100	Genetic	50	50
11	f	39 w 0 d	3840	241	100	Genetic	65	65
12	m	41 w 2 d	3070	251	100	Infection	43	43
13	m	40 w 0 d	3590	216	100	Genetic, NICU	55	55
14	f	41 w 0 d	3400	289	50 (German)	Genetic	85	85
15	f	38 w 0 d	3350	617	30 (Romanian)	Unknown	73	93
16	m	38 w 0 d	2680	434	100	Unknown	57	48
17	m	39 w 0 d	NA	320	40 (Arabic)	Genetic	105	103
18	m	37 w 0 d	2400	149	100	Genetic	94	94
19	f	30 w 1 d	1080	488	100	Prematurity, NICU	60	60
20	f	39 w 1 d	2950	273	100	Genetic	40	40
21	f	40 w 1 d	3200	161	20 (LIS)	Unknown	>90	>90
22	f	40 w 0 d	2650	341	100	Genetic	75	75
23	m	38 w 5 d	3200	238	100	Genetic	46	46
24	m	40 w 0 d	3350	249	100	Unknown	47	47
25	f	36 w 0 d	2700	230	100	Genetic	65	65
26	f	40 w 0 d	3600	255	50 (Ukraine)	Genetic, infection	55	55
27	m	37 w 5 d	2980	245	100	Unknown	45	45
28	m	37 w 4 d	3670	293	100	Genetic	75	75
29	m	38 w 0 d	3090	196	100	Genetic	60	60
30	m	38 w 0 d	2980	167	90 (Russian)	Unknown	63	63
31	f	38 w 0 d	2950	177	100	Genetic	64	64
32	f	37 w 0 d	2200	231	100	Unknown	95	95
33	m	37 w 0 d	2700	282	90 (Romanian)	NICU, antibiotics	82	87

LIS, lingua dei segni italiana (Italian sign language).

As deaf infants constitute a highly variable population, differing in the cause of deafness, language experience, age, etc., and this high variability has important theoretical and clinical implications, we have also decided to assess the HRF of several subgroups in our sample. Specifically, we chose to investigate the HRFs of monolingual deaf infants, genetically deaf infants, and deaf infants aged 5 to 12 months because in our sample and more generally, these profiles are frequent; thus, relatively large samples could be analyzed. Furthermore and more importantly, all three factors, i.e., language experience, cause of deafness, and age, are related to specific changes in neural development and brain plasticity. For instance, multilingualism is linked to more prolonged neuroplasticity in young infants,^45^^,^^46^ whereas perceptual narrowing and the underlying closure of critical periods for the phonology of the native language take place approximately during the second half of the first year of life.^45^^,^^47^^,^^48^ Therefore, in addition to the whole sample, data from three clinically and theoretically relevant subsets of the full sample were also analyzed separately: (i) monolingual Italian infants (n=27), standardly defined^49^^–^^51^ as receiving at least 80% exposure to Italian, (ii) genetically deaf infants, who constitute the most frequent etiology in our sample (n=17), (iii) infants between 5 and 12 months of age, the most common age for implantation, accounting for the majority of our sample (n=24).

A group of 72 typically developing and typically hearing infants were also recruited and tested as controls at the Audiology Service of the Otolaryngology Ward of the University Hospital of Padua tested. Of these, 25 infants could not be included in the final analysis due to poor data quality (12), fussiness or crying (4), the interruption of the experiment (5), and not accepting the cap/testing (4). Data from the remaining 47 infants (mean age: 145 days; age range: 9 to 382 days; 22 females) were entered into the final analysis. All infants were exposed to Italian at least 20% of the time, and nine of them were also exposed to a second language (Russian: 1 infant 50% exposure; Albanian: 1 infant 70%, 1 infant 40%; Portuguese: 1 infant 50%; Romanian: 1 infant 80%; English: 1 infant 10%, Moldavian: 2 infants both 50%; Arabic: 1 infant 70%). We note that the typically hearing infants were not matched in age but rather in medical history to the deaf infants as they were referred to the Audiology Service of the Otolaryngology Ward of the University Hospital of Padua for a suspicion of hearing loss, but upon clinical examination, have proven to have typical hearing. Thus, they followed the same initial medical trajectory and were tested under exactly the same circumstances as the deaf infants. Just as with deaf infants, we also analyzed the HRF characteristics of two subgroups of infants in the typically hearing population: (i) monolingual Italian infants (n=39; note that although nine infants were exposed to a second language other than Italian, in the case of one such infant, the amount of exposure to the second language was 10%, this infant thus still qualifies as monolingual), and (ii) infants between 5 and 12 months of age (n=20).

Parents or legal guardians of all infants gave written informed consent prior to participation. The study was approved by the Ethics Committees of the Padua and Naples Hospitals (IRB numbers: 5267/AO/21 and 66/2023, respectively).

### 2.2 Stimuli

Four language conditions were tested in the study. Stimuli for the Forward Italian condition consisted of 16 Italian sentences produced by two female native speakers of Italian (eight sentences each). The Backward Italian condition consisted of the same 16 Italian sentences played backward, i.e., time reversed using the “reverse” function in the Praat software.^52^ Backward speech is used as a nonlinguistic control stimulus as it matches forward speech in all nontemporal characteristics (e.g., intensity and voice quality). The forward English condition consisted of 16 sentences spoken by two American English female native speakers (eight sentences each). The backward English condition consisted of the same 16 sentences played backward. The sentences varied among affirmative, interrogative, and exclamative sentence types and contained 12 syllables in both languages. The sentences were produced in mild infant-directed speech. They did not differ significantly in duration, pitch, or intensity between the conditions [analyses of variance with factors Language (Italian vs. English) and direction (FW vs. BW), all ns]. Further details of the stimuli are reported here.^53^

The stimuli were presented in a total of 16 blocks, four blocks per condition [Fig. 1(c)]. Every block comprised four sentences. No sentence was repeated during the study. The order of the blocks was intermixed and counterbalanced across participants. No more than two blocks of the same condition occurred consecutively. Blocks were separated by silent intervals jittered between 15 and 18 s. Within blocks, sentences were also separated by silences jittered between 400 and 600 ms, yielding blocks of ∼12 to 13 s. The whole experiment lasted ∼8  min. To ensure sufficient data even when the infant participant did not complete the whole experiment, e.g., due to fussiness or crying, we organized the stimulus presentation such that two blocks of each condition were presented during the first half of the study, whereas the other two blocks of each condition were presented during the second half of the study. In this way, a participant who completed only half of the study anyway heard at least two blocks of each condition.

### 2.3 Procedure

Infants were tested in a quiet room at the Audiological Service of the Otolaryngology Ward Service of the University Hospital of Padua or at the Hearing and Balance Unit of the University Hospital of Naples, in the presence of at least one of their parents. During the study, infants were seated on a parent’s lap while their brain responses were measured with a NIRx NIRSport2 8–8 machine (source–detector separation: 3 cm; pulsated LED lights at two wavelengths of 760 and 850 nm; sampling rate: 20.345 Hz). The optical probes were inserted into a stretchy cap (EasyCap, Brain-Products GmbH, Gilching, Germany) placed on the infants’ head using surface landmarks (nasion and the preauricular points), covering the language areas in the bilateral temporal, frontal, and parietal cortices [10 channels/hemisphere; Fig. 1(b)]. The size of the cap was chosen based on each infant’s head circumference. To localize the 20 channels, we conducted an anatomical localization analysis,^54^ projecting channel positions relative to surface landmarks onto the cortical surface of 3D age-appropriate anatomical head scans, following standard practices in the infant NIRS field.^54^^–^^57^ Channels 1, 2, 4, and 5 in the LH and channels 13, 14, 15, and 16 in the RH were on average positioned over the frontal area, channels 3 and 6 in the LH and channels 17 and 19 in the RH over the temporal area, channel 9 in the LH and channel 21 in the RH were positioned in the frontier between the temporal and parietal areas, and channels 7, 10, and 12 in the LH and channels 18, 20, and 23 in the RH were on average positioned over the parietal area. Stimuli were presented using E-Prime and delivered through two speakers placed at an angle of 30 deg on the two sides and ∼1  m from the infant’s head. The stimuli were presented at a conversational intensity of around 65 dB. The computer running E-Prime sent time stamps to the NIRS machine. To keep infants’ attention during the study, they watched a silent cartoon on a computer screen played in front of them.

### 2.4 Data Preprocessing

To have the most faithful estimate possible of the HRF, we opted for a conservative pre-processing pipeline that has been shown to minimize the introduction of artifacts and other modifications to the underlying HRF and is routinely used in infant studies.^58^ In particular, we avoided using artifact correction and other data enhancement algorithms.

Accordingly, fNIRS light intensity measures were first converted to optical densities. Optical densities were then converted to oxygenated (oxyHb) and deoxygenated (deoxyHb) concentration changes, using the modified Beer–Lambert law, using the following absorption coefficients (μa, mM−1×mm−1): μa (oxyHb, 760 nm) = 0.1496, μa (oxyHb, 850 nm) = 0.2526, μa (deoxyHb, 760 nm) = 0.3865, and μa (deoxyHb, 850 nm) = 0.1798. The product of the optical pathlength and the differential pathlength factor was set to 1 so that the resulting concentration changes are expressed in mM × mm. Data were then band-pass filtered using a digital fft (fast Fourier transform) filter, between 0.01 and 0.7 Hz. Finally, a block in a given channel was rejected if the light intensity reached the saturation value (1.2 V), if the block contained a motion artifact, or both. Motion artifacts were defined as signal changes larger than 0.1 mM × mm over two samples. Finally, for the nonrejected blocks, a baseline was linearly fit between the means of the 5 s preceding the onset of the block and the 5 s starting 23 s after the onset of the block (13 s of stimulation plus 10 s of resting period).

### 2.5 Estimation of the HRF

To characterize the HRF, we extracted block-averaged data from the channels covering the bilateral temporal, i.e., auditory and speech processing areas, where effects are observed in this paradigm.^35^^,^^38^^–^^42^ The left temporal area included channels 3 and 6, the right channels 17 and 19, as identified using a localization analysis.^54^^,^^56^^,^^59^ Data from all the nonrejected blocks of the Forward Italian condition were averaged together within a given infant and across the two channels in each hemisphere to improve the signal-to-noise ratio and obtain a robust response for each child in each hemisphere. We then used a model-based, parameter fitting approach^4^^,^^60^ on these data.

We first estimated individual participants’ HRF parameters, separately for oxygenated and deoxygenated hemoglobin (oxyHb and deoxyHb, respectively), fitting them via nonlinear least-squares optimization to a triple gamma function (1). Specifically, we fit each infant’s NIRS response curve using constrained nonlinear least-squares optimization. A parametric hemodynamic response model composed of three gamma-shaped components (early dip, main peak, and undershoot) is fitted to the data, minimizing the sum of squared residuals between the measured signal and the model, with a capped number of function evaluations to balance accuracy and computational efficiency. To account for uncertainty in response polarity, the optimization is run twice with opposite sign constraints (canonical and inverted), and the solution with the higher coefficient of determination ($R^{2}$) is retained as the final fit. We used the default shape parameters of SPM ($n_{\text{dip}}=3$; $n_{\text{peak}}=6$; $n_{\text{undersh}}=16$).^61^

$$y(t)=A_{\text{dip}}\times g(t-t_{0};n_{\text{dip}},\tau \times s)+A_{\text{peak}}\times g(t-t_{0};n_{\text{peak}},\tau \times s)+A_{\text{undersh}}\times g(t-t_{0};n_{\text{undersh}},\tau \times s)$$ (1)

where

gamma kernel $g(t;n,\tau )=\{\begin{array}{ll} \frac{t^{n-1}e^{-\frac{t}{\tau }}}{\tau^{n}\times \Gamma (n)}, & t>0\\ 0, & t\leq 0 \end{array}$

A: amplitude of the initial dip

A: amplitude of the main peak

A: amplitude of undershoot

$t_{0}$: response onset delay

n: shape parameters ($n_{\text{dip}}=3$; $n_{\text{peak}}=6$; $n_{\text{undersh}}=16$)

τ: base time constant

s: scaling factor (stretch/compression)

Γ(n): gamma function for normalization

For each participant, the fitted curve was used to estimate the main peak amplitude, time-to-peak (TTP), and full width at half maximum (FWHM), as well as an initial dip (minimum value prior to the main peak) and a final undershoot (minimum value after the main peak). For both the dip and the undershoot, the amplitude and latency were recorded if the deflection exceeded 5% of the main peak amplitude. The HRF was allowed to take the canonical form, i.e., optional negative initial dip, positive peak, and optional negative undershoot, or an inverted response, i.e., optional positive initial dip, negative peak, and optional positive undershoot. Although its exact causes and interpretation are not fully understood yet, the inverted response is common in infant NIRS studies.^7^^,^^9^^,^^62^ Model performance, i.e., goodness-of-fit, was quantified using the coefficient of determination ($R^{2}$), calculated from the residual sum of squares relative to the total variance. Finally, we computed the group-level average and variance of the parameters.

### 2.6 Unit-Like HRF Reconstruction

In addition to the above described HRF estimate, we also implemented another approach to estimate the unit-like infant HRF independently of the characteristics of the experimental design. The fitted gamma functions reflect infants’ responses to the block design of the study, which is the most commonly used paradigm in infant NIRS. In such a response, individual responses to the single stimulus presentations within a block add up, providing a more robust response, the time course of which, however, is shaped by the design of the specific experiment (e.g., block duration). It is, therefore, also of relevance to reconstruct the unit-like response, which could then be used as a starting point for the data analysis of studies with designs quite different from the one used here. To achieve this, we followed Minagawa-Kawai et al.’s (2011) approach,^63^ who estimated the HRF of young infants, i.e., the population of interest of the current study. Specifically, we used a finite impulse response (FIR)-based estimation. The mean NIRS response of each group was submitted to a GLM with 20 FIR regressors. Each regressor was a 1-s-long rectangular pulse starting at 0, 1, 2, …, 20 s with respect to block onset. The number of regressors was chosen to be 20, following Minagawa-Kawai et al. (2011), as the infant hemodynamic response is known to be slower than the adult response,^62^^,^^64^^–^^66^ and a time window of 20 s can readily capture the full latency of this slower response, including undershoots and return to baseline. The beta coefficients were then extracted from the GLM for each FIR regressor, defining the curve for the estimated unit-like HRF. The estimation was done separately for oxyHb and deoxy in each group.

### 2.7 Statistical Analysis

We ran different statistical comparisons. First, we statistically compared parameter values between the left and the right hemispheres (LH & RH, respectively) in each group, using paired sample t-tests for each parameter.

In addition, we compared the HRF parameters of deaf infants with those of the typically hearing babies using a mixed ANOVA with between-subject factor group (deaf/typical hearing) and within-subject factor (hemisphere) for each HRF parameter.

Further, in deaf infants, we correlated the amplitude of the main peak in oxyHb with the hearing threshold of the contralateral ear to assess whether variability in the HRFs may be directly related to hearing loss. We also ran multiple linear regression analyses over the amplitude and time-to-peak latency of the mean peak in the LH and the RH separately with the demographic variables reported in Table 1 as predictors. Importantly, here, we focus on the characterization of the HRF in deaf and hard-of-hearing infants here; we thus did not conduct a similar analysis for the typical group.

## 3 Results

We estimated infants’ HRF parameters in response to their native language, forward Italian, i.e., the language condition that is familiar to them through their residual auditory experience, and that serves as the input for language acquisition. We first analyzed the HRF parameters for the typically hearing infants, including the full sample as well as the monolingual subsample and the subsample of infants aged 5 to 12 months, then the full sample of deaf infants, and the three clinically relevant subsamples, monolingual Italian-exposed deaf infants, infants with genetic deafness, and deaf infants between 5 and 12 months of age.

### 3.1 Typically Hearing Infants

#### 3.1.1 Full sample

##### OxyHb

**Fig. 2:**
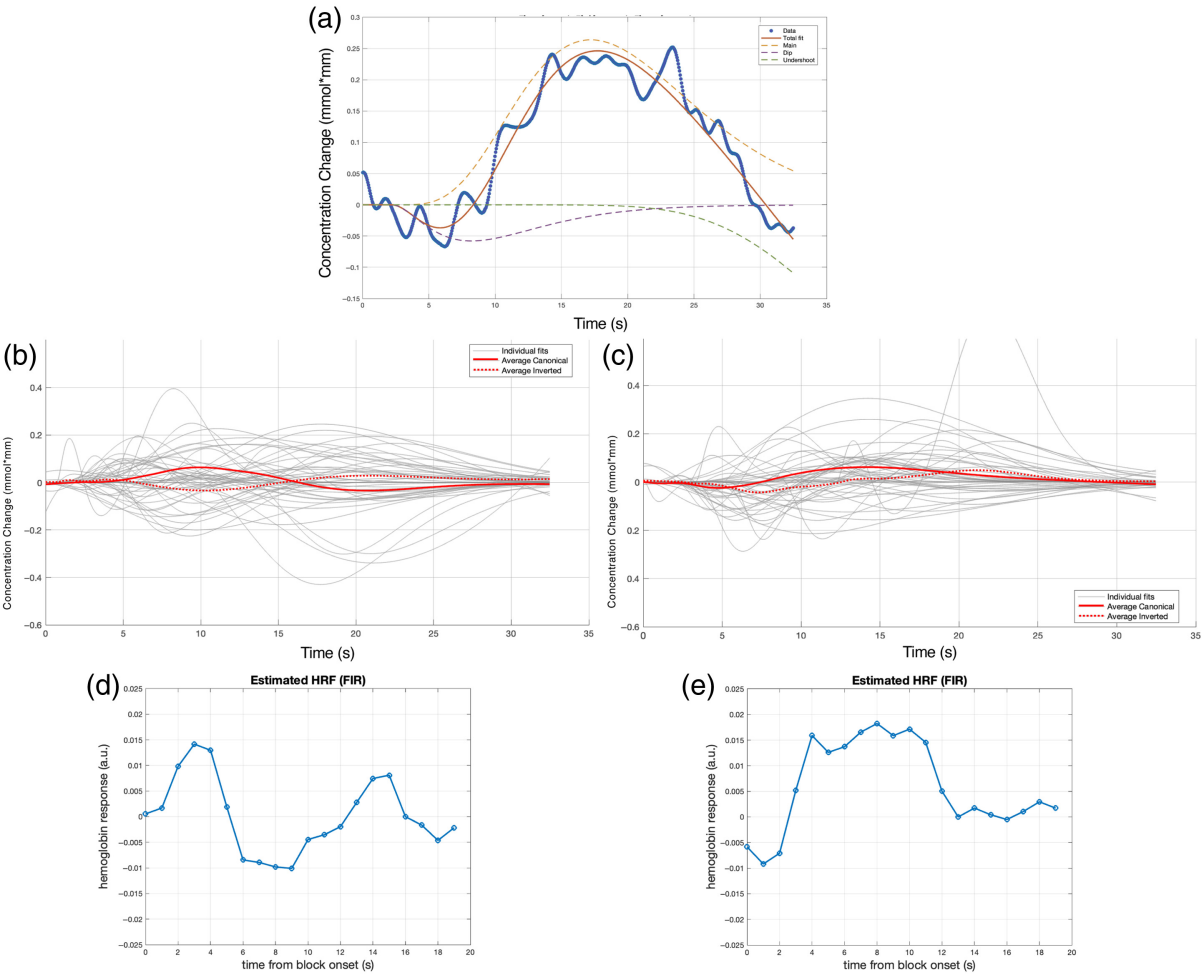
(a) Example of fitting over the HRF of a single participant. (b), (c) The average fitted gamma functions of the typically hearing infants in response to the forward Italian condition in the left (b) and right (c) temporal areas. The reconstructed unit HRF in the left (d) and right (e) temporal areas.

**Fig. 3:**
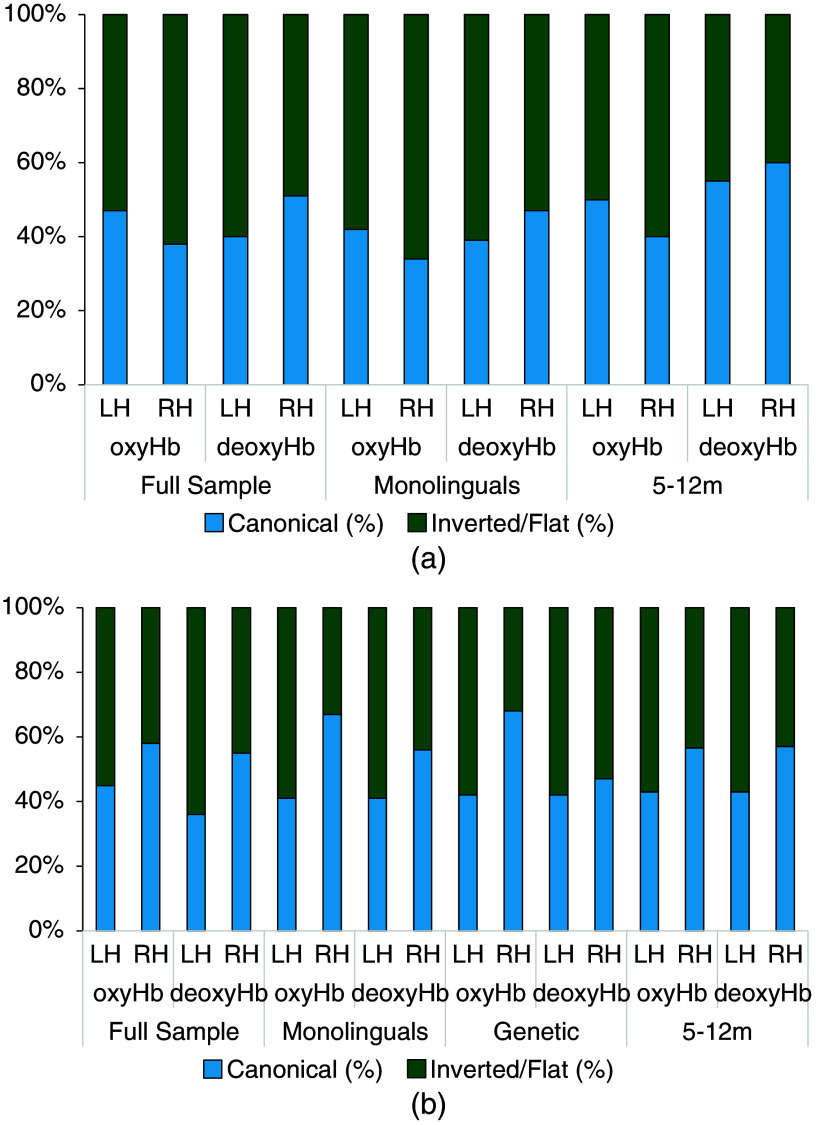
(a) Percentages of canonical and inverted hemodynamic responses in the full sample of typically hearing infants as well as in the monolingual and 5- to 12-month-old subgroups. (b) The percentages of canonical and inverted hemodynamic responses in the full deaf sample and the three clinically relevant subgroups.

**Fig. 4:**
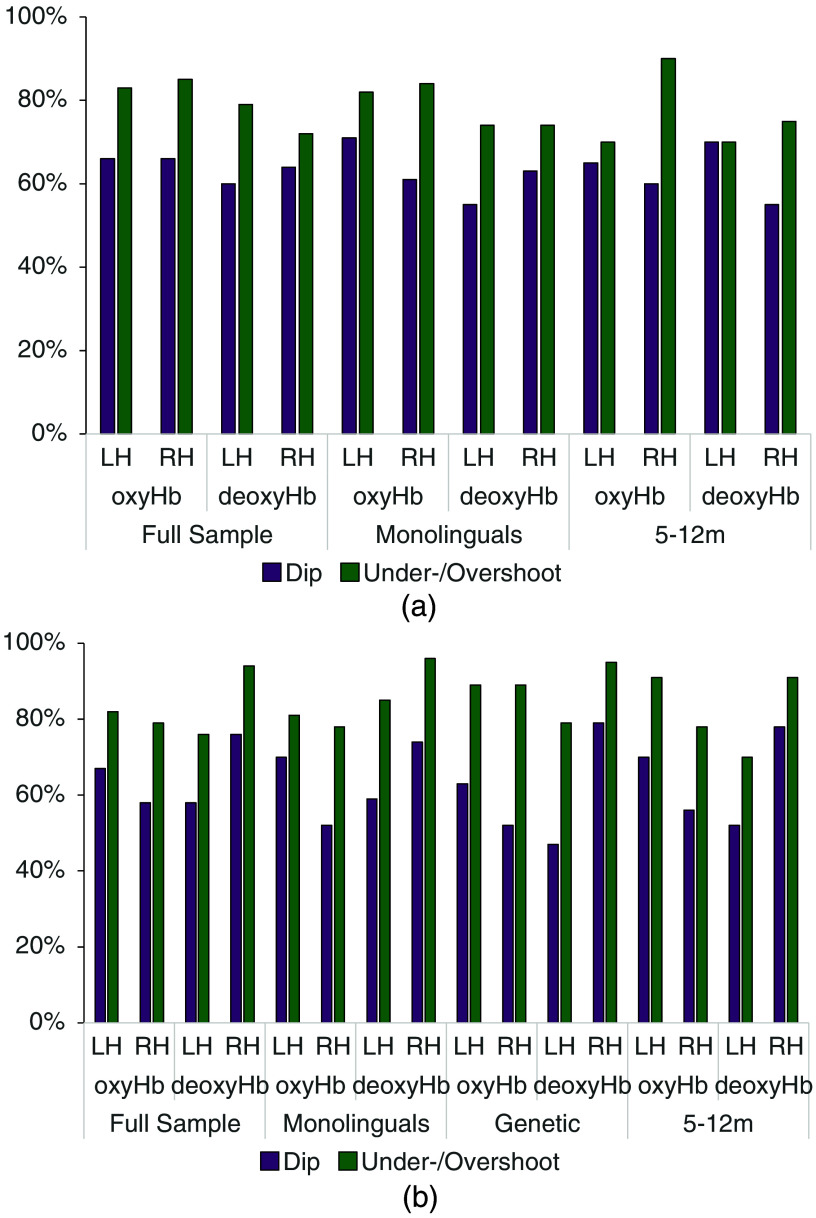
(a) Percentages of infants showing an initial dip or an under-/overshoot in the full sample of typically hearing infants as well as in the monolingual and 5- to 12-month-old subsample. (b) The percentages of infants showing an initial dip or an under-/overshoot in the full sample of deaf infants and the three clinically relevant subgroups.

The fit for an individual infant is illustrated in Fig. 2(a) as an example. The individual and the average fitted gamma functions for the left temporal area (channels 3 and 6) are shown in Fig. 2(b). Of the 47 infants, 22 (47%) infants produced a canonical hemodynamic response, i.e., with a positive peak, 25 (53%) showed an inverted hemodynamic response, i.e., a negative peak [Fig. 3(a)]. The initial dip was present (negative in infants with a canonical HRF; positive in infants with an inverted HRF) in 31 infants (66%), and the final undershoot was present (negative in infants with a canonical HRF; positive in infants with an inverted HRF) in 39 infants [83%; Fig. 4(a)]. Table 2 summarizes the HRF parameters.

**Table 2:** Estimated oxyHb HRF parameters (A) and their standard deviations (B) for forward Italian.

Typically hearing infants’ averages		Left hemisphere										Right hemisphere									
Group	HRF shape	Peak amplitude	Time-to-peak	FWHM	Initial dip amplitude	Dip latency	Final under-shoot	Under-shoot latency	Response delay	Scaling factor	Good-ness-of-fit	Peak amplitude	Time-to-peak	FWHM	Initial dip amplitude	Dip latency	Final under-shoot	Under-shoot latency	Response delay	Scaling factor	Good-ness-of-fit
A																					
**Full sample**	All	−0,002	10,065	9,023	0,022	4,763	0,015	19,559	1,699	1,532	0,648	−0,025	10,806	9,886	0,012	5,460	0,055	17,326	2,549	1,455	0,586
	Canonical	0,121	10,626	9,174	−0,060	4,890	−0,110	19,988	1,538	1,676	0,696	0,100	11,670	9,641	−0,069	5,195	−0,063	19,184	1,561	1,817	0,592
	Inverted	−0,110	9,556	8,949	0,089	4,651	0,123	19,232	1,840	1,406	0,606	−0,102	10,254	9,750	0,064	5,624	0,134	16,438	3,162	1,231	0,582
**Monolingual**	All	−0,005	9,716	9,335	0,032	4,382	0,035	19,650	1,378	1,502	0,628	−0,027	10,515	9,862	0,030	5,207	0,070	17,342	2,317	1,445	0,613
	Canonical	0,120	10,978	9,726	−0,063	4,700	−0,102	20,006	1,192	1,754	0,631	0,103	11,540	9,451	−0,060	4,898	−0,061	18,804	1,336	1,781	0,570
	Inverted	−0,096	8,786	9,034	0,089	4,152	0,122	19,454	1,513	1,319	0,625	−0,103	9,930	9,773!	0,078	5,388	0,149	16,649	2,890	1,249	0,638
**5 to 12 months**	All	−0,015	10,541	9,020	0,016	5,055	−0,008	19,580	1,910	1,572	0,616	−0,020	10,525	9,048	0,002	6,125	0,085	18,084	3,651	1,237	0,522
	Canonical	0,111	10,816	9,156	−0,079	4,394	−0,108	18,100	1,175	1,610	0,628	0,088	10,643	8,924	−0,075	5,598	−0,046	21,557	2,234	1,682	0,505
	Inverted	−0,141	10,320	9,006	0,098	5,715	0,091	20,874	2,645	1,535	0,603	−0,092	10,431	9,177!	0,056	6,477	0,169	16,190	4,595	0,941	0,534
Typically hearing infants’ SDs		Left hemisphere										Right hemisphere									
Group	HRF shape	Peak amplitude	Time-to-peak	FWHM	Initial dip amplitude	Dip latency	Final under-shoot	Under-shoot latency	Response delay	Scaling factor	Good-ness-of-fit	Peak amplitude	Time-to-peak	FWHM	Initial dip amplitude	Dip latency	Final under-shoot	Under-shoot latency	Response delay	Scaling factor	Good-ness-of-fit
B																					
**Full sample**	All	0,141	3,633	4,332	0,091	2,653	0,157	4,956	2,605	0,768	0,250	0,133	3,480	4,825	0,090	3,229	0,148	4,908	3,825	0,938	0,292
	Canonical	0,067	3,593	4,490	0,043	2,851	0,115	4,978	3,073	0,862	0,265	0,097	3,203	5,066	0,051	2,098	0,046	6,543	2,120	0,955	0,259
	Inverted	0,091	3,677	4,219	0,059	2,520	0,096	5,036	2,168	0,667	0,233	0,085	3,599	4,803!	0,068	3,793	0,141	3,757	4,504	0,869	0,315
**Monolingual**	All	0,130	3,051	4,229	0,093	2,478	0,146	5,324	2,692	0,752	0,252	0,135	3,372	4,956	0,091	3,102	0,159	5,108	3,712	0,929	0,225
	Canonical	0,061	3,549	4,688	0,048	3,149	0,098	6,014	3,470	0,910	0,283	0,105	3,333	5,264	0,046	2,275	0,052	7,251	2,223	0,992	0,260
	Inverted	0,081	2,303	4,175	0,059	1,898	0,095	5,061	2,032	0,568	0,233	0,083	3,331	4,621	0,070	3,531	0,149	3,768	4,294	0,850	0,203
**5 to 12 months**	All	0,152	3,667	4,932	0,107	3,143	0,131	4,430	3,306	0,807	0,272	0,134	3,931	4,552	0,090	3,658	0,186	5,319	4,129	0,835	0,344
	Canonical	0,055	2,654	4,932	0,052	3,460	0,089	5,272	4,147	0,950	0,338	0,112	3,446	4,683	0,045	1,458	0,029	6,015	1,849	0,882	0,196
	Inverted	0,102	4,450	#DIV/0!	0,062	2,813	0,080	3,364	2,161	0,684	0,204	0,094	4,464	4,318	0,072	4,629	0,197	4,000	4,984	0,685	0,424

The individual and average fitted gamma functions for the right temporal area (channels 17 and 19) are shown in Fig. 2(c). Of the 47 infants, 18 (38%) infants produced a canonical hemodynamic response, i.e., with a positive peak, 29 (62%) showed an inverted hemodynamic response, i.e., a negative peak [Fig. 3(a)]. The initial dip was present (negative in infants with a canonical HRF; positive in infants with an inverted HRF) in 31 (66%) infants, and the final undershoot was present (negative in infants with a canonical HRF; positive in infants with an inverted HRF) in 40 [85%; Fig. 4(a)] infants. Table 2 summarizes the HRF parameters.

The paired sample t-tests for the different parameters revealed no significant differences between the LH and the RH (all |t|<2, ns.).

The FIR-based estimate is illustrated in Figs. 2(d) and 2(e), respectively, for the LH and RH. In the LH, the unit HRF shows a peak at 3 s after stimulus onset, reaching the maximum of the undershoot at 9 s, an overshoot at 15 s, followed by a return to baseline. In the RH, the unit HRF had an initial dip peaking at 1 s, reached its main peak at 8 s, showed no clear undershoot and returned to baseline at 13 s.

##### DeoxyHb

**Fig. 5:**
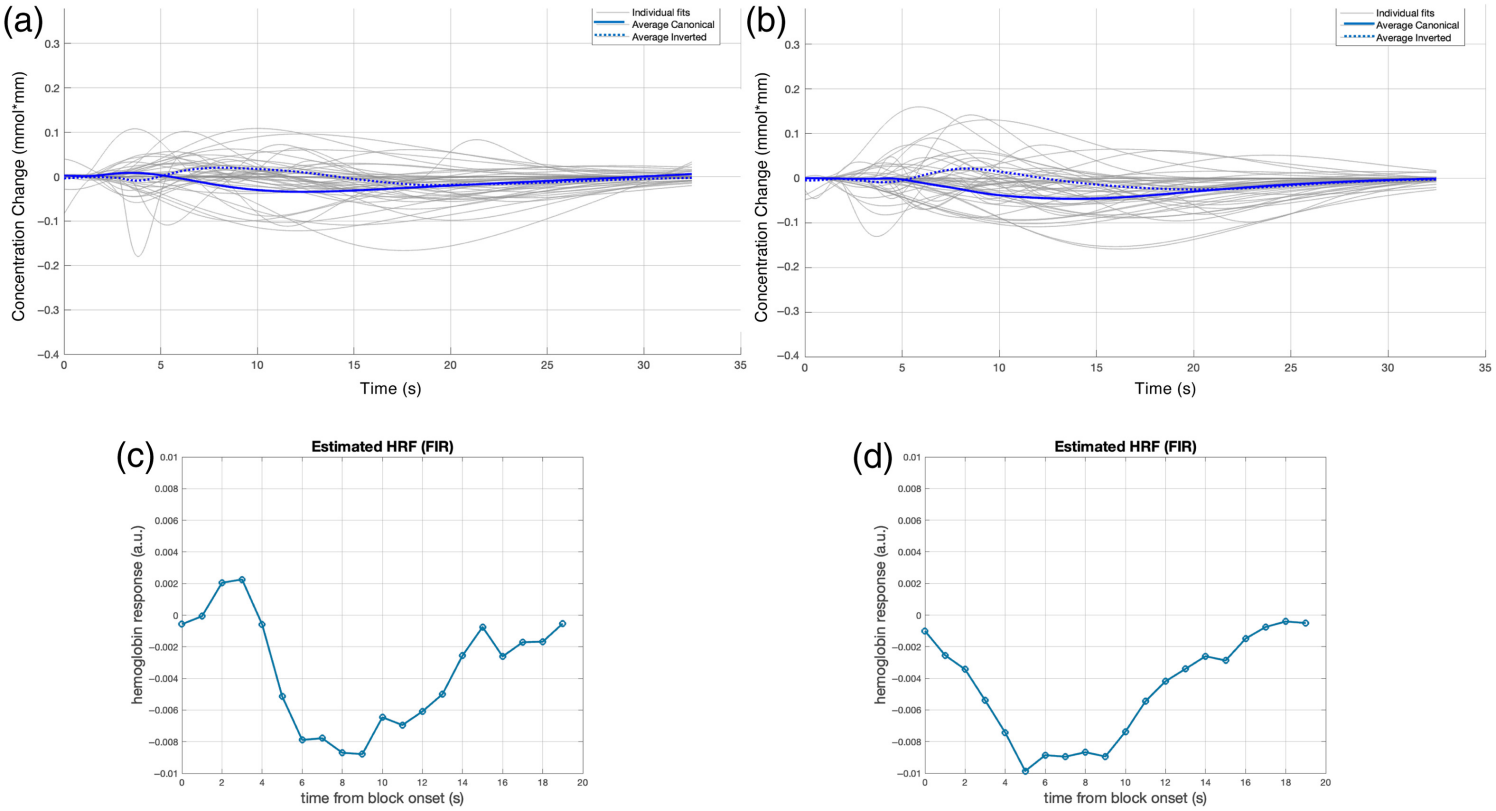
Average HRF and fitted gamma functions for the deoxyHb responses of typically hearing infants in response to the forward Italian condition in the left (a) and right (b) temporal areas. The reconstructed unit HRF in the left (c) and right (d) temporal areas.

The individual and the average fitted gamma functions for the left temporal area (channels 3 and 6) are shown in Fig. 5(a). Nineteen (40%) of the 47 infants produced a canonical hemodynamic response, i.e., with a negative deoxyHb peak, 28 (60%) showed a flat (close to zero) or an inverted response, i.e., a positive deoxyHb peak (Fig. 3). The initial dip was present (positive in infants with a canonical HRF; negative in infants with an inverted HRF) in 28 (60%) infants, the final overshoot (positive in infants with a canonical HRF; negative in infants with an inverted HRF) in 37 (79%) infants (Fig. 4). Table 3 summarizes the HRF parameters.

**Table 3:** Estimated deoxyHb HRF parameters (A) and their standard deviations (B) for forward Italian in the sample of typically hearing infants.

Typically hearing infants’ averages		Left hemisphere										Right hemisphere									
Group	HRF shape	Peak amplitude	Time-to-peak	FWHM	Initial dip amplitude	Dip latency	Final under-shoot	Under-shoot latency	Response delay	Scaling factor	Good-ness-of-fit	Peak amplitude	Time-to-peak	FWHM	Initial dip amplitude	Dip latency	Final under-shoot	Under-shoot latency	Response delay	Scaling factor	Good-ness-of-fit
A																					
**Full sample**	All	0,005	10,320	6,226	−0,014	5,096	−0,016	20,008	1,956	1,570	0,563	−0,010	10,756	5,774	−0,021	5,057	−0,019	18,719	1,844	1,607	0,611
	Canonical	−0,054	12,386	5,994!	0,044	4,988	0,031	22,533	0,732	2,128	0,619	−0,067	12,044	5,638	0,028	4,984	0,032	19,358	0,824	2,080	0,618
	Inverted	0,045	9,019	6,433	−0,042	5,170	−0,041	19,036	2,786	1,192	0,525	0,048	9,339	5,907	−0,049	5,134	−0,047	18,429	2,907	1,113	0,604
**Monolingual**	All	0,008	10,325	6,656	−0,025	4,958	−0,019	19,963	1,803	1,578	0,548	−0,012	10,921	5,984	−0,026	5,167	−0,024	18,937	1,940	1,613	0,609
	Canonical	−0,056	12,028	6,878	0,029	4,301	0,027	22,664	−0,027	2,164	0,604	−0,074	12,353	6,062	0,030	4,743	0,028	19,337	0,423	2,160	0,615
	Inverted	0,050	9,319	6,476	−0,047	5,386	−0,041	18,934	2,996	1,195	0,512	0,044	9,647	5,950	−0,050	5,548	−0,048	18,790	3,305	1,121	0,604
**5 to 12 months**	All	−0,019	11,686	5,995	−0,001	5,827	−0,001	19,360	2,244	1,791	0,518	−0,010	10,911	6,221	−0,025	4,914	−0,011	19,104	1,515	1,700	0,570
	Canonical	−0,061	13,685	5,762	0,038	5,638	0,040	21,976	0,983	2,328	0,603	−0,055	11,923	5,871	0,031	5,046	0,035	21,107	1,040	2,003	0,633
	Inverted	0,031	9,466	6,027	−0,024	6,058	−0,036	18,053	3,786	1,136	0,413	0,057	9,320	6,606	−0,067	4,716	−0,046	17,387	2,226	1,245	0,475
Typically hearing infants’ SDs		Left hemisphere										Right hemisphere									
Group	HRF shape	Peak amplitude	Time-to-peak	FWHM	Initial dip amplitude	Dip latency	Final under-shoot	Under-shoot latency	Response delay	Scaling factor	Good-ness-of-fit	Peak amplitude	Time-to-peak	FWHM	Initial dip amplitude	Dip latency	Final under-shoot	Under-shoot latency	Response delay	Scaling factor	Good-ness-of-fit
B																					
**Full sample**	All	0,063	4,216	3,208	0,060	3,614	0,043	5,992	3,862	0,830	0,238	0,073	3,159	2,459	0,048	2,712	0,046	5,887	3,091	0,876	0,210
	Canonical	0,050	4,577	3,114!	0,037	3,503	0,019	7,584	2,899	0,810	0,256	0,041	3,060	2,231	0,026	2,306	0,024	7,888	2,398	0,882	0,228
	Inverted	0,031	3,457	3,230	0,049	3,749	0,029	5,102	4,248	0,607	0,222	0,048	2,675	2,871	0,031	3,131	0,026	4,921	3,412	0,541	0,193
**Monolingual**	All	0,066	3,744	3,477	0,057	3,482	0,042	6,463	3,984	0,867	0,246	0,073	3,121	2,827	0,049	2,767	0,045	6,372	3,171	0,893	0,214
	Canonical	0,052	3,259	3,329	0,023	2,429	0,017	8,308	1,894	0,835	0,278	0,042	3,073	2,671	0,030	2,391	0,025	9,381	2,076	0,871	0,231
	Inverted	0,030	3,711	3,511	0,053	4,019	0,031	5,504	4,544	0,657	0,222	0,044	2,632	3,075	0,033	3,077	0,028	5,194	3,404	0,580	0,204
**5 to 12 months**	All	0,064	4,637	4,338	0,037	3,469	0,043	6,634	3,571	0,971	0,250	0,068	2,879	4,116	0,061	2,150	0,051	6,765	2,438	0,910	0,237
	Canonical	0,057	5,268	4,439	0,022	4,329	0,021	10,207	3,553	0,797	0,251	0,032	2,461	4,250	0,031	2,372	0,027	8,471	2,807	0,905	0,234
	Inverted	0,023	2,573	3,992	0,020	2,246	0,019	4,319	3,106	0,752	0,221	0,049	2,925	3,994	0,038	1,905	0,033	4,927	1,672	0,753	0,221

The individual and average fitted gamma functions for the right temporal area (channels 17 and 19) are shown in Fig. 5(b). Of the 47 infants, 24 (51%) produced a canonical response, i.e., with a negative peak, 23 (49%) a flat response, i.e., close to zero, or an inverted response, i.e., a positive peak. The initial dip was present (positive in infants with a canonical HRF; negative in infants with an inverted HRF) in 30 infants (64%), and the final overshoot was present (positive in infants with a canonical HRF; negative in infants with an inverted HRF) in 34 (72%) infants. Table 3 summarizes the HRF parameters.

The paired sample t-tests for the different parameters revealed no significant differences between the LH and the RH (all |t|<2, ns.).

The FIR-based reconstructed unit HRFs are shown in Figs. 5(c) and 5(d), for the LH and RH, respectively. In the LH, the HRF showed an initial positive dip with a peak at 3 s, reached its minimum at 9 s, and returned to baseline at 15 s. In the RH, the HRF showed no initial dip, reached its minimum at 5 s, and returned to baseline at 18 s.

#### 3.1.2 Monolingual typically hearing infants

##### OxyHb

For the left auditory area, the fit did not converge for 1 out of the 39 monolingual infants. Of the remaining 38, 16 (42%) infants produced a canonical hemodynamic response, and 22 (58%) showed an inverted response (Fig. 3). The initial dip was detected in 27 (71%) infants, and the final undershoot was detected in 31 (82%) infants (Fig. 4). Table 2 summarizes the HRF parameters.

For the right auditory area, the fit did not converge for 1 out of the 39 monolingual infants. Of the remaining 38, 14 (37%) infants showed a canonical hemodynamic response, and 24 (63%) showed an inverted response (Fig. 3). An initial dip could be identified in 23 (61%) infants, and the final undershoot could be identified in 32 (84%) infants (Fig. 4). Table 2 summarizes the HRF parameters.

##### DeoxyHb

For the left auditory area, the fit did not converge for 1 out of the 39 monolingual infants. Of the remaining 38 infants, 15 (39%) infants produced a canonical hemodynamic response, and 23 (61%) showed a flat (close to zero) or inverted response in the left auditory area (Fig. 3). The initial dip was detected in 21 (55%) infants, whereas the final overshoot was detected in 28 (74%) infants (Fig. 4). Table 3 summarizes the HRF parameters.

In the right auditory area, fitting did not converge for 1 infant. Of the remaining 38, 18 (47%) showed a canonical hemodynamic response, and 20 (53%) showed an inverted response (Fig. 3). An initial dip could be identified in 24 (63%) infants, and the final overshoot could be identified in 28 (74%) infants (Fig. 4). Table 3 summarizes the HRF parameters.

#### 3.1.3 Hearing infants aged 5 to 12 months

##### OxyHb

Of the 20 infants aged 5 to 12 months, 10 (50%) produced a canonical hemodynamic response, and 10 (50%) showed an inverted response (Fig. 3) in the left auditory area. The initial dip was detected in 13 (65%) infants, and the final undershoot was detected in 14 (70%) infants (Fig. 4). Table 2 summarizes the HRF parameters.

For the right auditory area, 8 (40%) of the 20 infants aged 5 to 12 months showed a canonical hemodynamic response, and 12 (60%) showed an inverted response (Fig. 3). An initial dip could be identified in 12 (60%) infants, and the final undershoot could be identified in 18 (90%) infants (Fig. 4). Table 2 summarizes the HRF parameters.

##### DeoxyHb

Of the 20 infants aged 5 to 12 months, 11 (55%) infants produced a canonical hemodynamic response, and 9 (45%) showed a flat (close to zero) or inverted response in the left auditory area (Fig. 3). The initial dip was detected in 11 (55%) infants, and the final overshoot was detected in 15 (75%) infants (Fig. 4). Table 3 summarizes the HRF parameters.

In the right auditory area, 12 (60%) out of the 20 infants between 5 and 12 months showed a canonical response, and 8 (40%) showed an inverted response (Fig. 3). An initial dip could be identified in 14 (70%) infants, and the final overshoot could be identified in 14 (70%) infants (Fig. 4). Table 3 summarizes the HRF parameters.

### 3.2 Deaf and Hard-of-Hearing Infants

#### 3.2.1 Full sample

##### OxyHb

**Fig. 6:**
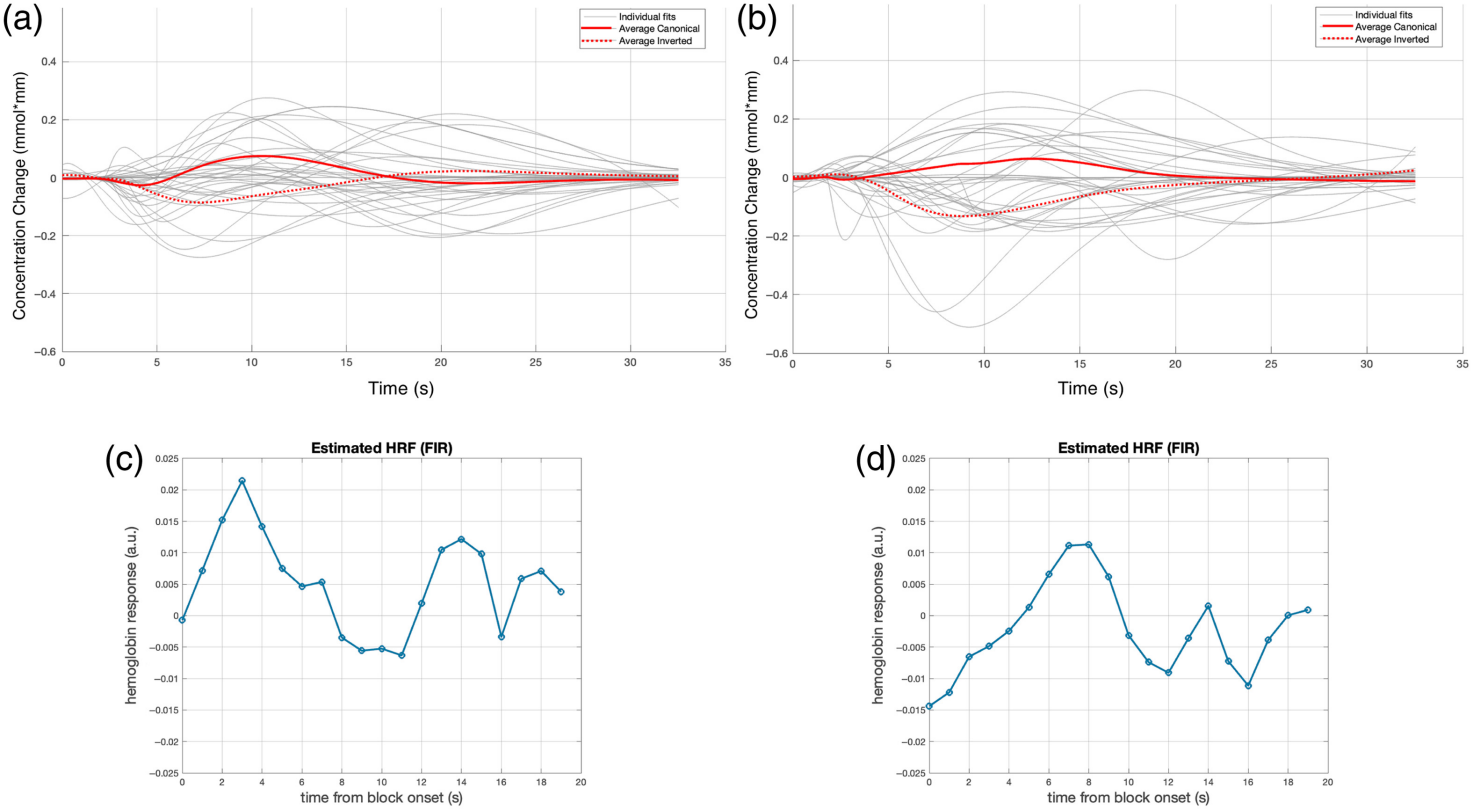
Average fitted gamma functions of all deaf infants in response to the forward Italian condition in the left (a) and right (b) temporal areas. The reconstructed HRF in the left (c) and right (d) temporal areas.

The individual and average fitted gamma functions for the left temporal area (channels 3 and 6) are shown in Fig. 6(a). Of the 33 infants, 15 (45%) produced a canonical hemodynamic response, i.e., with a positive peak, and 18 (55%) showed an inverted hemodynamic response, i.e., with a negative peak [Fig. 3(b)]. The initial dip was present (negative in infants with a canonical HRF; positive in infants with an inverted HRF) in 22 infants (67%), and the final undershoot was present (negative in infants with a canonical HRF; positive in infants with an inverted HRF) in 27 infants [82%; Fig. 4(b)]. Table 2 summarizes the HRF parameters.

The individual and average fitted gamma functions for the right temporal area (channels 17 and 19) are shown in Fig. 6(b). Of the 33 infants, 19 (58%) produced a canonical hemodynamic response, i.e., with a positive peak, and 14 (42%) showed an inverted hemodynamic response, i.e., with a negative peak [Fig. 3(b)]. The initial dip was present (negative in infants with a canonical HRF; positive in infants with an inverted HRF) in 19 (58%) infants, and the final undershoot was present (negative in infants with a canonical HRF; positive in infants with an inverted HRF) in 26 [79%; Fig. 4(b)] infants. Table 2 summarizes the HRF parameters. There was a statistically significant difference between the LH and the RH in the time-to-peak latency of the main peak (t(32)=−2.066; p=0.047), which occurred earlier in the LH than in the RH. No other difference between the two hemispheres was significant.

We found no correlation between the amplitude of the peak in the LH and the hearing threshold of the left (r=−0.020, ns.) or the right ear (r=−0.045, ns.), or the RH peak amplitude and the right (r=−0.103, ns.) or left (r=−0.153, ns.) hearing threshold. The multiple regression analyses over the amplitudes and latencies of the main peak in the LH and the RH revealed no significant predictors.

The FIR-based reconstructed HRFs are shown for the LH and RH, respectively, in Figs. 6(c) and 6(d). In the LH, the unit HRF reached its main peak at 3 s with no initial dip, followed by an undershoot with a peak at 11 s, and returned to baseline after multiple over- and undershoots at 19 s. In the RH, the HRF started with an initial dip over the first 4 s, reached its main peak at 8 s and returned to baseline after multiple under- and overshoots at 18 s.

The mixed ANOVAs with between-subject factor group (deaf/typically hearing) and within-subject factor (hemisphere) were not significant for any of the HRP parameters.

##### DeoxyHb

**Fig. 7:**
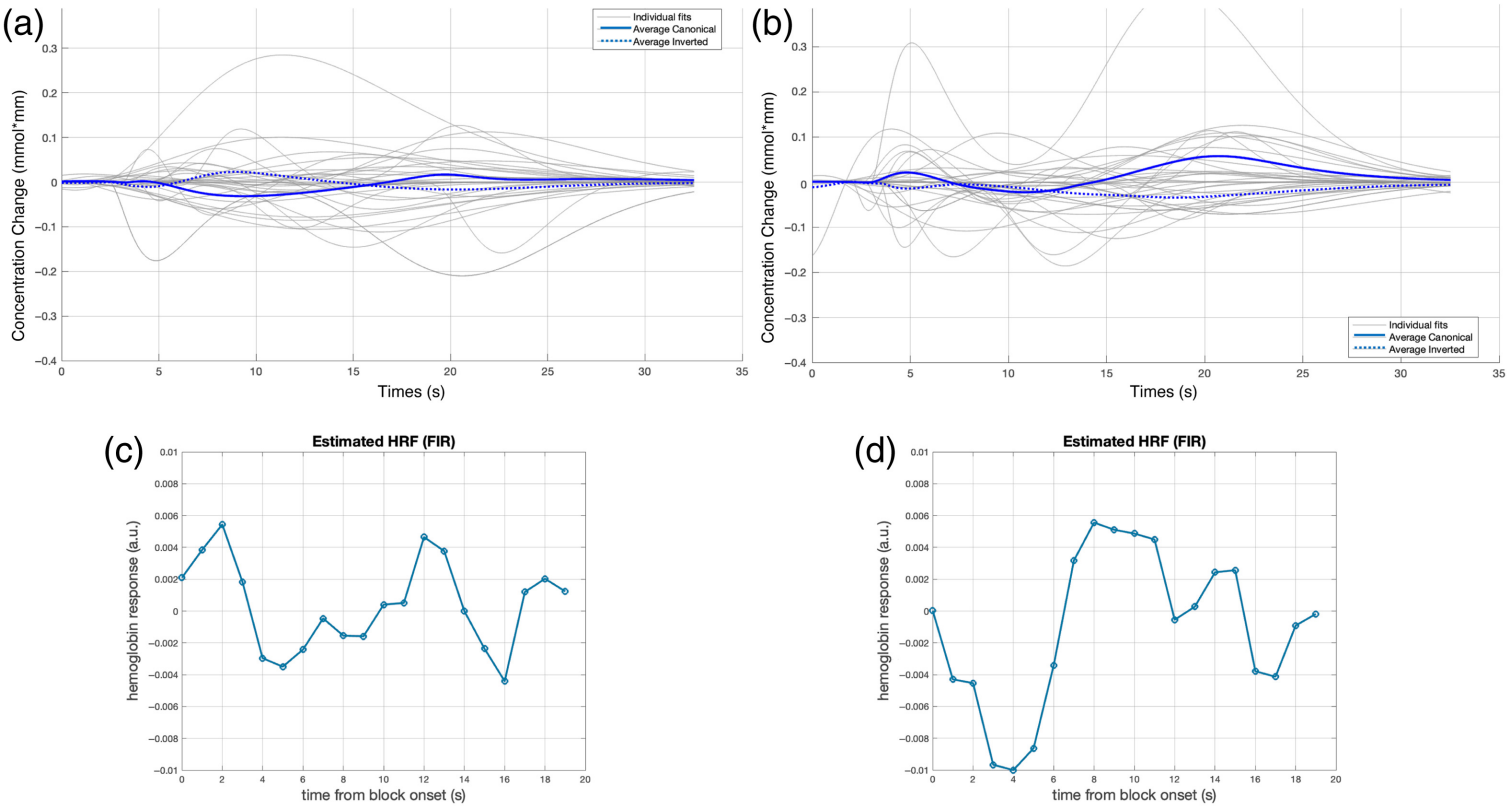
Average fitted gamma functions of all deaf infants in response to the forward Italian condition in the left (a) and right (b) temporal areas. The reconstructed HRF in the left (c) and right (d) temporal areas.

The individual and average fitted gamma functions for the left temporal area (channels 3 and 6) are shown in Fig. 7(a). Of the 33 infants, 12 (36%) of whom infants produced a canonical hemodynamic response, i.e., with a negative deoxyHb peak, 21 (64%) showed a flat (close to zero) or an inverted response, i.e., a positive deoxyHb peak (Fig. 3). The initial dip was present (positive in infants with a canonical HRF; negative in infants with an inverted HRF) in 19 (58%) infants, the final overshoot (positive in infants with a canonical HRF; negative in infants with an inverted HRF) in 25 (76%) infants. Table 3 summarizes the HRF parameters and shows the parameters that yielded a statistically significant difference between the LH and the RH.

The individual and average fitted gamma functions for the right temporal area (channels 17 and 19) are shown in Fig. 7(b). Of the 33 infants, 18 (55%) produced a canonical response, i.e., with a negative peak, and 15 (45%) produced a flat response, i.e., close to zero, or an inverted response, i.e., a positive peak. The initial dip was present (positive in infants with a canonical HRF; negative in infants with an inverted HRF) in 25 infants (76%), and the final overshoot was present (positive in infants with a canonical HRF; negative in infants with an inverted HRF) in 31 (94%) infants. Table 3 summarizes the HRF parameters. The paired sample t-tests showed a statistically significant difference between the LH and the RH in terms of the amplitude of the overshoot (t(23)=2.288, p=0.032), which was greater in the RH than in the LH. No other parameters differed statistically significantly between the LH and the RH, as shown by paired sample t-tests (all |t|<2, ns.).

The mixed ANOVA with between-subject factor group (deaf/typically hearing) and within-subject factor (hemisphere) over the amplitude values of the main peak showed a main effect of Hemisphere as the main peak was more negative, i.e., stronger, in the RH than in the LH in both groups (F(1,78)=3.879, p=0.050). All other main effects and interactions were nonsignificant. In a similar ANOVA over the amplitude of the overshoot, there was a main effect of hemisphere (F(1,50)=4.650, p=0.036) due to greater, i.e., more positive overshoots in the RH than in the LH, and a hemisphere × group interaction (F(1,50)=5.539, p=0.023), as the deaf group, but not the hearing group showed stronger overshoots in the RH than in the LH. The ANOVAs over the other HRF metrics did not yield significant main effects or interactions.

The reconstructed unit HRF is illustrated in Figs. 7(c) and 7(d) for the LH and RH, respectively. In the LH, the HRF starts with an initial positive dip peaking at 2 s, reaches its minimum at 5 s, shows an overshoot with a peak at 12 s, and returns to baseline at 17 s after an undershoot. In the RH, the HRF reaches its minimum at 4 s, overshoots with a maximum at 8 s, and returns to baseline through under- and overshoots at 19 s.

#### 3.2.2 Monolingual deaf infants

##### OxyHb

For the left auditory area, of the 27 monolingual infants, 11 (41%) infants produced a canonical hemodynamic response, and 16 (59%) showed an inverted response. The initial dip was detected in 19 (70%) infants, and the final undershoot was detected in 22 (81%) infants. Table 4 summarizes the HRF parameters.

**Table 4:** Estimated oxyHb HRF parameters (A) and their standard deviations (B) in the full deaf sample and subgroups.

Deaf infants’ averages		Left hemisphere										Right hemisphere									
Group	HRF shape	Peak amplitude	Time-to-peak	FWHM	Initial dip amplitude	Dip latency	Final under-shoot	Under-shoot latency	Response delay	Scaling factor	Good-ness-of-fit	Peak amplitude	Time-to-peak	FWHM	Initial dip amplitude	Dip latency	Final under-shoot	Under-shoot latency	Response delay	Scaling factor	Good-ness-of-fit
A																					
**Full sample**	All	−0,001	9,697	9,005	0,010	4,359	0,009	19,147	1,434	1,463	0,628	−0,019	11,260	9,173	−0,018	4,551	−0,016	20,055	0,902	1,825	0,517
	Canonical	0,112	10,379	8,727	−0,069	4,656	−0,124	20,005	1,511	1,573	0,651	0,109	11,067	8,994	−0,079	4,316	−0,096	19,584	0,926	1,695	0,492
	Inverted	−0,095	9,106	9,337	0,075	4,112	0,132	18,460	1,369	1,371	0,609	−0,191	11,483	9,221	0,049	4,871	0,111	20,822	0,868	2,001	0,550
**Monolingual**	All	−0,019	9,900	8,933	0,013	4,345	0,031	18,894	1,441	1,452	0,621	0,008	11,399	8,773	−0,031	4,596	−0,022	20,027	1,083	1,756	0,510
	Canonical	0,107	10,849	8,730	−0,069	4,753	−0,108	20,007	1,658	1,547	0,612	0,105	10,752	8,610	−0,079	4,196	−0,094	19,584	0,950	1,623	0,487
	Inverted	−0,105	9,243	9,101	0,073	4,065	0,146	18,122	1,291	1,387	0,627	−0,184	12,533	8,862	0,054	5,397	0,157	21,179	1,349	2,024	0,557
**Genetic**	All	0,008	9,948	8,842	0,020	4,537	0,009	19,322	1,516	1,510	0,667	0,011	11,221	8,789	−0,061	4,536	−0,050	20,584	0,928	1,804	0,529
	Canonical	0,123	10,226	8,744	−0,045	4,612	−0,140	19,734	1,316	1,648	0,679	0,087	11,341	8,542	−0,097	4,623	−0,106	19,939	1,404	1,610	0,462
	Inverted	−0,076	9,732	8,996	0,053	4,482	0,113	19,048	1,662	1,410	0,659	−0,153	11,021	8,914	0,025	4,347	0,087	22,733	−0,102	2,224	0,676
**5 to 12 months**	All	−0,010	9,118	8,449	0,020	4,250	0,008	18,581	1,669	1,291	0,643	−0,007	10,626	9,294	−0,016	3,755	−0,006	20,628	0,046	1,855	0,490
	Canonical	0,097	10,449	8,383	−0,064	4,494	−0,117	17,962	1,537	1,478	0,643	0,117	10,835	9,581	−0,090	3,646	−0,098	20,866	0,035	1,806	0,424
	Inverted	−0,093	8,150	8,692	0,086	4,063	0,122	18,962	1,771	1,146	0,643	−0,168	10,394	8,889!	0,047	3,895	0,108	20,272	0,059	1,918	0,575
Deaf infants’ SDs		Left hemisphere										Right hemisphere									
Group	HRF shape	Peak amplitude	Time-to-peak	FWHM	Initial dip amplitude	Dip latency	Final under-shoot	Under-shoot latency	Response delay	Scaling factor	Good-ness-of-fit	Peak amplitude	Time-to-peak	FWHM	Initial dip amplitude	Dip latency	Final under-shoot	Under-shoot latency	Response delay	Scaling factor	Good-ness-of-fit
B																					
**Full sample**	All	0,127	3,233	4,427	0,090	2,329	0,175	5,668	2,200	0,733	0,263	0,189	3,616	4,396	0,084	2,988	0,131	6,932	3,102	0,895	0,297
	Canonical	0,064	3,322	4,169	0,045	2,473	0,133	5,773	2,308	0,807	0,303	0,091	2,204	4,857	0,067	2,789	0,079	7,613	3,379	0,915	0,324
	Inverted	0,079	3,145	4,337	0,057	2,243	0,105	5,686	2,172	0,674	0,231	0,144	4,864	4,017	0,030	3,318	0,090	6,071	2,807	0,869	0,266
**Monolingual**	All	0,128	3,454	4,428	0,090	2,528	0,167	6,221	2,269	0,746	0,275	0,176	3,592	4,606	0,089	3,225	0,142	7,250	3,304	0,881	0,297
	Canonical	0,066	3,565	4,161	0,051	2,810	0,107	6,671	2,334	0,812	0,347	0,092	1,902	4,691	0,071	2,819	0,082	7,613	3,475	0,884	0,332
	Inverted	0,078	3,356	4,529	0,059	2,368	0,108	6,040	2,287	0,716	0,225	0,145	5,447	4,559	0,034	3,980	0,087	6,872	3,114	0,860	0,222
**Genetic**	All	0,119	3,163	4,363	0,056	2,183	0,173	4,352	2,284	0,760	0,209	0,129	2,839	4,886	0,085	2,688	0,131	7,246	3,210	0,944	0,345
	Canonical	0,054	3,725	4,492	0,013	2,391	0,163	2,380	2,524	0,847	0,173	0,070	2,469	5,066	0,074	3,124	0,087	8,035	3,691	0,955	0,364
	Inverted	0,071	2,868	4,227	0,036	2,137	0,079	5,422	2,208	0,715	0,240	0,032	3,623	4,491	0,008	1,587	0,120	3,931	1,599	0,843	0,270
**5 to 12 months**	All	0,117	3,086	4,384	0,095	2,374	0,173	5,191	2,142	0,632	0,286	0,180	2,587	4,023	0,090	1,758	0,132	7,794	1,751	0,855	0,320
	Canonical	0,056	4,146	4,322	0,050	3,008	0,140	4,970	2,374	0,832	0,355	0,080	2,174	4,578	0,078	1,933	0,071	8,691	1,903	0,863	0,345
	Inverted	0,077	1,630	4,487	0,063	1,859	0,110	5,486	2,039	0,400	0,235	0,140	3,103	3,998	0,032	1,591	0,094	7,000	1,633	0,886	0,280

For the right auditory area, 18 (67%) of the 27 monolingual infants showed a canonical hemodynamic response, and 9 (33%) showed an inverted response. An initial dip could be identified in 14 (52%) infants, and the final undershoot could be identified in 21 (78%) infants. Table 4 summarizes the HRF parameters.

The mixed ANOVA with between-subject factor group (deaf/typically hearing) and within-subject factor (hemisphere) over the amplitude values of the final undershoot also showed a main effect of group as the final undershoot was more negative in the deaf than in the hearing group (F(1,32)=4.214, p=0.048). All other main effects and interactions were nonsignificant. Similar ANOVAs for the other HRF parameters showed no significant results or interactions.

##### DeoxyHb

Of the 27 monolingual infants, 11 (41%) produced a canonical hemodynamic response, and 16 (59%) showed a flat (close to zero) or inverted response in the left auditory area. The initial dip was detected in 16 (59%) infants, whereas the final overshoot was detected in 23 (85%) infants. Table 5 summarizes the HRF parameters.

**Table 5:** Estimated deoxyHb HRF parameters (A) and their standard deviations (B) for forward Italian in the full deaf sample and subgroups.

Deaf infants’ averages		Left hemisphere										Right hemisphere									
Group	HRF shape	Peak amplitude	Time-to-peak	FWHM	Initial dip amplitude	Dip latency	Final under-shoot	Under-shoot latency	Response delay	Scaling factor	Good-ness-of-fit	Peak amplitude	Time-to-peak	FWHM	Initial dip amplitude	Dip latency	Final under-shoot	Under-shoot latency	Response delay	Scaling factor	Good-ness-of-fit
A																					
**Full sample**	All	0,010	10,470	8,583	−0,018	5,163	−0,023	18,934	2,010	1,576	0,581	−0,014	10,718	7,563	−0,005	5,602	0,016	19,492	2,887	1,357	0,604
	Canonical	−0,055	11,033	8,870	0,033	6,078	0,055	22,859	2,774	1,652	0,495	−0,053	11,100	8,285!	0,052	6,458	0,087	20,456	3,696	1,381	0,599
	Inverted	0,047	10,094	8,134	−0,042	4,640	−0,067	16,411	1,574	1,533	0,630	0,032	10,129	7,051	−0,067	4,574	−0,071	18,378	1,916	1,329	0,609
**Monolingual**	All	−0,003	10,565	8,136	−0,018	5,312	−0,023	19,082	2,289	1,512	0,594	−0,011	11,540	7,253	−0,006	6,014	0,012	19,106	3,282	1,366	0,617
	Canonical	−0,050	10,855	8,392	0,033	6,267	0,055	22,859	3,208	1,529	0,519	−0,068	11,289	7,865	0,010	6,306	0,005	27,900	2,984	1,661	0,912
	Inverted	0,029	10,320	7,958	−0,049	4,656	−0,072	15,992	1,656	1,500	0,646	0,035	11,008	6,816	−0,079	4,900	−0,074	17,038	2,332	1,284	0,641
**Genetic**	All	−0,004	9,612	7,854	−0,010	4,628	−0,012	18,453	1,524	1,552	0,583	−0,014	11,065	8,293	0,000	5,446	0,004	20,316	2,381	1,533	0,606
	Canonical	−0,050	9,871	8,203	0,036	4,919	0,071	21,251	1,617	1,651	0,562	−0,059	11,198	8,509	0,032	6,574	0,057	21,566	3,491	1,541	0,620
	Inverted	0,030	9,405	7,531	−0,047	4,416	−0,068	16,355	1,456	1,480	0,598	0,026	10,894	8,093	−0,035	4,431	−0,049	19,222	1,381	1,525	0,594
**5 to 12 months**	All	−0,002	9,744	7,639	−0,023	4,585	−0,038	19,368	1,492	1,546	0,602	−0,015	10,287	6,116	−0,019	4,985	0,007	19,686	2,388	1,299	0,586
	Canonical	−0,066	11,221	7,852	0,036	5,938	0,045	23,351	2,415	1,761	0,496	−0,052	11,374	5,909	0,029	6,468	0,070	20,986	3,671	1,398	0,603
	Inverted	0,047	8,401	7,325	−0,065	3,544	−0,102	16,580	0,783	1,380	0,683	0,034	8,112	6,204	−0,079	3,058	−0,077	18,257	0,720	1,169	0,563
Deaf infants’ SDs		Left hemisphere										Right hemisphere									
Group	HRF shape	Peak amplitude	Time-to-peak	FWHM	Initial dip amplitude	Dip latency	Final under-shoot	Under-shoot latency	Response delay	Scaling factor	Good-ness-of-fit	Peak amplitude	Time-to-peak	FWHM	Initial dip amplitude	Dip latency	Final under-shoot	Under-shoot latency	Response delay	Scaling factor	Good-ness-of-fit
B																					
**Full sample**	All	0,079	3,891	4,731	0,063	3,610	0,085	5,305	4,228	0,873	0,216	0,064	3,221	4,174	0,093	3,784	0,112	4,315	4,450	0,780	0,208
	Canonical	0,052	4,432	4,512	0,028	5,130	0,044	4,801	6,149	0,840	0,266	0,051	3,235	4,209	0,083	4,024	0,100	4,209	4,933	0,830	0,213
	Inverted	0,067	3,570	4,824	0,061	2,364	0,069	3,991	2,692	0,909	0,169	0,045	3,261	3,941	0,057	3,315	0,046	4,327	3,722	0,743	0,210
**Monolingual**	All	0,057	4,132	5,092	0,069	3,886	0,088	5,485	4,414	0,852	0,203	0,062	3,129	4,228	0,102	4,033	0,119	4,621	4,776	0,856	0,197
	Canonical	0,051	4,603	5,122	0,028	5,336	0,044	4,801	6,254	0,760	0,265	0,046	3,051	4,102	0,089	4,259	0,110	4,508	5,329	0,901	0,222
	Inverted	0,033	3,864	4,959	0,069	2,464	0,072	3,922	2,581	0,935	0,134	0,050	3,403	4,396	0,060	3,594	0,049	4,033	4,000	0,829	0,168
**Genetic**	All	0,052	3,440	4,883	0,071	2,599	0,093	4,681	3,214	0,888	0,241	0,059	3,090	4,579	0,044	3,512	0,067	3,702	4,303	0,847	0,220
	Canonical	0,045	2,975	4,729	0,034	3,367	0,046	4,070	4,564	0,885	0,290	0,050	2,916	4,603	0,028	3,367	0,049	2,424	4,624	0,903	0,191
	Inverted	0,023	3,919	4,913	0,074	2,026	0,071	4,138	1,999	0,926	0,212	0,030	3,531	4,472	0,029	3,488	0,026	4,409	3,961	0,843	0,254
**5 to 12 months**	All	0,089	3,793	4,192	0,079	3,671	0,097	5,677	4,262	0,801	0,188	0,065	3,176	3,304	0,071	3,860	0,088	4,801	4,339	0,656	0,233
	Canonical	0,049	4,464	4,295	0,030	5,004	0,040	5,414	5,980	0,855	0,225	0,047	3,312	3,602!	0,030	4,261	0,048	4,683	5,084	0,817	0,238
	Inverted	0,081	2,585	3,976	0,077	1,796	0,075	4,096	2,286	0,749	0,101	0,053	1,289	1,911	0,061	2,215	0,048	4,746	2,467	0,360	0,237

In the right auditory area, 15 (56%) of the 27 monolingual infants showed a canonical hemodynamic response, and 12 (44%) showed an inverted response. An initial dip could be identified in 20 (74%) infants, and the final overshoot could be identified in 26 (96%) infants. Table 5 summarizes the HRF parameters.

The mixed ANOVA with the between-subject factor group (deaf/typically hearing) and within-subject factor (hemisphere) over the amplitude of the final overshoot yielded a significant hemisphere × group interaction (F(1,42)=4.256, p=0.045), as the deaf group, but not the hearing group showed stronger overshoots in the RH than in the LH. All other main effects and interactions were nonsignificant. None of the ANOVAs over the other HRF metrics yielded significant main effects or interactions.

#### 3.2.3 Genetically deaf infants

##### OxyHb

For the left auditory area, out of the 19 genetically deaf infants, 8 (42%) infants produced a canonical hemodynamic response, and 11 (58%) showed an inverted response. The initial dip was detected in 12 (63%) infants, and the final undershoot was detected in 17 (89%) infants. Table 4 summarizes the HRF parameters.

For the right auditory area, of the 19 infants, 13 (68%) showed a canonical hemodynamic response, and 6 (32%) showed an inverted response. An initial dip could be identified in 10 (53%) infants, and the final undershoot could be identified in 17 (89%) infants. Table 4 summarizes the HRF parameters.

##### DeoxyHb

Of the 19 genetically deaf infants, 8 (42%) produced a canonical hemodynamic response, and 11 (58%) showed a flat (close to zero) or inverted response in the left auditory area. The initial dip was detected in 9 (47%) infants, and the final overshoot was detected in 15 (79%) infants. Table 5 summarizes the HRF parameters.

In the right auditory area, 9 (47%) of the 19 genetically deaf infants showed a canonical response, and 10 (53%) showed an inverted response. An initial dip could be identified in 15 (79%) infants, and the final overshoot could be identified in 18 infants (95%). Table 5 summarizes the HRF parameters.

#### 3.2.4 Deaf infants aged 5 to 12 months

##### OxyHb

For the left auditory area, 10 (43%) of the 23 infants aged 5 to 12 months produced a canonical hemodynamic response, and 13 (57%) showed an inverted response. The initial dip was detected in 16 (70%) infants, and the final undershoot was detected in 21 (91%) infants. Table 4 summarizes the HRF parameters.

For the right auditory area, 13 (56%) of the 23 infants showed a canonical hemodynamic response, and 10 (43%) showed an inverted response. An initial dip could be identified in 31 (56%) infants, and the final undershoot could be identified in 18 (78%) infants. Table 4 summarizes the HRF parameters.

The mixed ANOVAs with between-subject factor group (deaf/typically hearing) and within-subject factor (hemisphere) over the latency of the initial dip yielded a significant main effect of group (F(1,41)=7.027, p=0.011) as the typically hearing infants had a longer dip latency than the deaf group. Similar ANOVAs over the other HRF metrics did not yield significant main effects or interactions.

##### DeoxyHb

Of the 23 infants aged 5 to 12 months, 10 (43%) produced a canonical hemodynamic response, and 13 (57%) showed a flat (close to zero) or inverted response in the left auditory area. The initial dip was detected in 12 (52%) infants, and the final overshoot was detected in 16 (70%) infants. Table 5 summarizes the HRF parameters.

In the right auditory area, 13 (57%) out of the 23 infants between 5 and 12 months showed a canonical response, and 10 (43%) showed an inverted response. An initial dip could be identified in 18 (78%) infants, and the final overshoot could be identified in 21 (91%) infants. Table 5 summarizes the HRF parameters.

The mixed ANOVAs with between-subject factor group (deaf/typically hearing) and within-subject factor (hemisphere) did not yield significant main effects or interactions for any of the HRF metrics.

## 4 Discussion

The current study characterizes the shape of the hemodynamic response function (HRF) measured with fNIRS in the bilateral auditory cortices of deaf and hard-of-hearing infants while they listened to Italian—the native language of most participants—using a simple block design. In addition, the study reconstructs the unit HRF for these groups. We have successfully obtained HRFs in most infants, indicating that in many cases, residual hearing is sufficient to allow infants to perceive at least some aspects of speech stimuli, as also found in previous studies.^28^

Deaf infants’ HRF has been found to peak around 9 to 12 s after stimulus onset and reach the same main peak amplitude as hearing infants’ HRF. It overall shows a similar shape as infant HRFs observed in our typical sample, with a few exceptions, as well as in other NIRS studies with young infants investigating speech perception.^35^^,^^38^^,^^41^^,^^67^^–^^70^ Specifically, deoxyHb showed no difference between the two groups in most parameters, except for a hemisphere × group interaction. This was due to the fact that the full deaf group and the monolingual deaf subgroup showed stronger deoxyHb overshoot in the RH than in the LH, whereas this hemispheric difference was not present in the typically hearing group. OxyHb also showed only minor differences between the two groups. The full samples were not different in any of the HRF parameters. The monolingual subgroups differed in the amplitude of the final undershoot, which was more negative in deaf infants. The 5- to 12-month-old subgroups, by contrast, differed in latency of the initial dip, which was longer in typically hearing than in deaf infants. Despite these differences, the responses were overall quite similar in the two groups. Finding similar HRFs between deaf and hearing infants is important because this result suggests that previous studies with deaf infants that relied on typical HRFs in their data analysis can be regarded as valid.

In addition to the main peak, most infants showed an initial dip and/or a final under-/overshoot in both hemispheres for both hemoglobin species. The former was somewhat less common, with ∼40% to 80% of infants exhibiting it. The latter was more frequent, with 60% to 90% of the infants showing it. These results suggest that when modelling the HRF of deaf infants, a function allowing for a double or triple lobed response is more appropriate than a function with a single peak. This was true for the full sample as well as for the subgroups we analyzed both in the deaf and the typically hearing populations.

In addition, as is common in the infant literature,^9^^,^^62^ we have found the oxyHb peak to be greater in amplitude than the deoxyHb peak, partly deriving from the fact that deoxyHb responses were more flat in several infants. This asymmetry is the reason why infant studies often find statistically more robust results with oxyHb as compared with deoxyHb. We nevertheless recommend reporting and statistically testing both hemoglobin species as deoxyHb responses still showed well-characterizable patterns with specific parameter values.

The reconstructed unit HRFs were also similar between the two groups. Interestingly, the most important difference could be observed between the two hemispheres, similarly in both groups, because the oxyHb response peaked earlier in the LH than in the RH in both groups. Although more research will be needed to understand the reasons for this difference, e.g., with nonlinguistic stimuli, we hypothesize that the earlier LH peak may be related to the left hemispheric lateralization of speech and language processing, triggering a faster response in this hemisphere.

Behind these general trends, we also discovered great interindividual variation. Indeed, the distribution of infants who showed a canonical response or an inverted one is approximately half and half (40% to 60%) across both hemispheres and hemoglobin species. The reasons for and possible interpretations of inverted hemodynamic responses remain debated,^7^^,^^9^ with neural maturation, task demands, and stimulus complexity being the most common factors in typical infants. As our sample consisted of atypical children, further variability may derive from the infants’ clinical conditions, although there was no direct correlation between infants’ response amplitudes and their hearing thresholds, and infants with an established genetic etiology showed HRFs that were similar to those of the full sample. Although our results mainly highlight similarities between deaf and typically hearing infants as groups, further research will be needed to understand individual variation in HRFs and HRF characteristics of subgroups that were not assessed in the current study, e.g., infants with different ages, language backgrounds, and etiologies.

Overall, responses in the left and right auditory cortices showed a similar shape, but we did find a statistically significant difference between the time-to-peak latency of the peak response, which was slower in the right than in the left hemisphere for oxyHb (but not for deoxyHb). This difference may be a reflection of the overall left lateralization of language functions already observable in the newborn brain for the native language.^35^^,^^38^ The LH, being more specialized in speech and language processing, may show a faster response.

A more detailed characterization of the HRF in typical and even more importantly in atypical populations can lead to better, more accurate imaging studies that can reveal how the developing brain is shaped by experience or lack thereof. For instance, studies assessing the time course of the hemodynamic response over the duration of the study, such as habituation to stimuli, or investigating connectivity may strongly benefit from a more HRF model. Furthermore, using more accurate HRFs, clinical studies could better assess whether differences in infants’ responses, say between the auditory and other sensory modalities is related to cortical reorganization due to deprivation or simply to underlying differences between HRF characteristics among different brain areas or in response to different tasks.

A limitation of the current study is that we did not compare HRF characteristics across channels covering similar brain areas as a way to validate our approach and results. Rather, we chose to average across the two channels by hemisphere that are known to reliably cover the bilateral temporal areas to obtain a more robust brain response. However, future studies could address this question, comparing HRF characteristics across channels.

## 5 Conclusion

Our results provide, for the first time, a detailed characterization of the hemodynamic response function in deaf and hard-of-hearing infants in response to speech in the native language. These results are of utmost importance for neuroimaging research in this atypical population and may considerably advance not only our knowledge of the brain of deaf infants but also therapeutic and clinical choices as they make it possible to analyze fMRI and fNIRS data in this population more accurately.

## Data Availability

The raw data that support the findings of this article are not publicly available as they are sensitive clinical data. Derived, preprocessed data, which were used for fitting, can be found at https://osf.io/8g9hy/?view_only=5660facd5c4447b98fc7eac7563fbeb2.

## References

[1] Glover G. H., “Deconvolution of impulse response in event-related BOLD fMRI,” NeuroImage 9(4), 416–429 (1999). NEIMEF 1053-8119 10.1006/nimg.1998.0419 10191170

[r2] Boynton G. M. et al., “Linear systems analysis of functional magnetic resonance imaging in human V1,” J. Neurosci. 16(13), 4207–4221 (1996). JNRSDS 0270-6474 10.1523/JNEUROSCI.16-13-04207.1996 8753882 PMC6579007

[r3] Plichta M. M. et al., “Model-based analysis of rapid event-related functional near-infrared spectroscopy (NIRS) data: a parametric validation study,” NeuroImage 35(2), 625–634 (2007). NEIMEF 1053-8119 10.1016/j.neuroimage.2006.11.028 17258472

[4] Handwerker D. A. Ollinger J. M. D’Esposito M., “Variation of BOLD hemodynamic responses across subjects and brain regions and their effects on statistical analyses,” NeuroImage 21(4), 1639–1651 (2004). NEIMEF 1053-8119 10.1016/j.neuroimage.2003.11.029 15050587

[5] Vrancken S. L. van Heijst A. F. de Boode W. P., “Neonatal hemodynamics: from developmental physiology to comprehensive monitoring,” Front. Pediatr. 6, 87 (2018). 10.3389/fped.2018.00087 29675404 PMC5895966

[r6] de Roever I. et al., “Investigation of the pattern of the hemodynamic response as measured by functional near-infrared spectroscopy (fNIRS) studies in newborns, less than a month old: a systematic review,” Front. Hum. Neurosci. 12, 371 (2018). 10.3389/fnhum.2018.00371 30333736 PMC6176492

[r7] Issard C. Gervain J., “Variability of the hemodynamic response in infants: influence of experimental design and stimulus complexity,” Dev. Cogn. Neurosci. 33, 182–193 (2018). 10.1016/j.dcn.2018.01.009 29397345 PMC6969282

[r8] Arichi T. et al., “Development of BOLD signal hemodynamic responses in the human brain,” NeuroImage 63(2), 663–673 (2012). NEIMEF 1053-8119 10.1016/j.neuroimage.2012.06.054 22776460 PMC3459097

[9] Gervain J. et al., “Using functional near-infrared spectroscopy to study the early developing brain: future directions and new challenges,” Neurophotonics 10(2), 023519 (2023). 10.1117/1.NPh.10.2.023519 37020727 PMC10068680

[10] Sharma A. Dorman M., “Central auditory system development and plasticity after cochlear implantation,” in Auditory Prostheses, Zeng F. G. Popper A. Fay R., Eds., pp. 233–255, Springer (2011).

[r11] Kral A. Sharma A., “Developmental neuroplasticity after cochlear implantation,” Trends Neurosci. 35(2), 111–122 (2012). TNSCDR 0166-2236 10.1016/j.tins.2011.09.004 22104561 PMC3561718

[r12] Sharma A. Dorman M. F. Spahr A. J., “Rapid development of cortical auditory evoked potentials after early cochlear implantation,” Neuroreport 13(10), 1365–1368 (2002). NERPEZ 0959-4965 10.1097/00001756-200207190-00030 12151804

[r13] Kral A. et al., “Hearing after congenital deafness: central auditory plasticity and sensory deprivation,” Cereb. Cortex 12(8), 797–807 (2002). 53OPAV 1047-3211 10.1093/cercor/12.8.797 12122028

[14] Gordon K. A. Papsin B. C. Harrison R. V., “Activity-dependent developmental plasticity of the auditory brain stem in children who use cochlear implants,” Ear Hear. 24(6), 485–500 (2003). 10.1097/01.AUD.0000100203.65990.D4 14663348

[15] Alencar C. D. C. Butler B. E. Lomber S. G., “What and how the deaf brain sees,” J. Cogn. Neurosci. 31(8), 1091–1109 (2019). JCONEO 0898-929X 10.1162/jocn_a_01425 31112472

[16] Pénicaud S. et al., “Structural brain changes linked to delayed first language acquisition in congenitally deaf individuals,” NeuroImage 66, 42–49 (2013). NEIMEF 1053-8119 10.1016/j.neuroimage.2012.09.076 23063844

[17] Bortfeld H., “Functional near-infrared spectroscopy as a tool for assessing speech and spoken language processing in pediatric and adult cochlear implant users,” Dev. Psychobiol. 61(3), 430–443 (2019). DEPBA5 0012-1630 10.1002/dev.21818 30588618 PMC7363196

[18] Saliba J. et al., “Functional near-infrared spectroscopy for neuroimaging in cochlear implant recipients,” Hear. Res. 338, 64–75 (2016). HERED3 0378-5955 10.1016/j.heares.2016.02.005 26883143 PMC4967399

[19] Anderson C. A. et al., “Adaptive benefit of cross-modal plasticity following cochlear implantation in deaf adults,” Proc. Natl. Acad. Sci. U.S.A. 114(38), 10256–10261 (2017). PNASA6 0027-8424 10.1073/pnas.1704785114 28808014 PMC5617272

[r20] Alemi R. et al., “Audiovisual integration in children with cochlear implants revealed through EEG and fNIRS,” Brain Res. Bull. 205, 110817 (2023). BRBUDU 0361-9230 10.1016/j.brainresbull.2023.110817 37989460

[r21] Deroche M. et al., “Cross-modal plasticity in children with cochlear implant: converging evidence from EEG and fNIRS” (2023). 10.1093/braincomms/fcae175 PMC11154148 38846536

[r22] Basura G. J. et al., “Human central auditory plasticity: a review of functional near‐infrared spectroscopy (fNIRS) to measure cochlear implant performance and tinnitus perception,” Laryngoscope Investig. Otolaryngol. 3(6), 463–472 (2018). 10.1002/lio2.185 PMC6302720 30599031

[r23] Chen Z. et al., “Individualized post‐operative prediction of cochlear implantation outcomes in children with prelingual deafness using functional near‐infrared spectroscopy,” Laryngoscope Investig. Otolaryngol. 9(6), e70035 (2024). 10.1002/lio2.70035 PMC11558700 39539355

[r24] Alemi R. et al., “Motor processing in children with cochlear implants as assessed by functional near-infrared spectroscopy,” Percept. Mot. Skills 131(1), 74–105 (2024). PMOSAZ 0031-5125 10.1177/00315125231213167 37977135 PMC10863375

[r25] Tseng H.-C. et al., “Predicting variability in pediatric cochlear implant outcomes through synchronous brain activation patterns: insights from fNIRS,” Hear. Res. 465, 109347 (2025). HERED3 0378-5955 10.1016/j.heares.2025.109347 40614488

[r26] Yamazaki H. et al., “Sensitivity and reliability of fNIRS to detect cochlear implant-induced auditory cortical activation in prelingually deaf children with inner ear malformation or cochlear nerve deficiency,” Brain Res. 1856, 149578 (2025). BRREAP 0006-8993 10.1016/j.brainres.2025.149578 40113194

[r27] Wang Y. et al., “The neural processing of vocal emotion after hearing reconstruction in prelingual deaf children: a functional near-infrared spectroscopy brain imaging study,” Front. Neurosci. 15, 705741 (2021).1662-453X 10.3389/fnins.2021.705741 34393716 PMC8355545

[r28] Coëz A. et al., “The recognition of human voice in deaf and hearing infants,” Hear. Balance Commun. 20(3), 179–185 (2022). 10.1080/21695717.2022.2084866

[r29] Harrison S. C. et al., “Use of functional near-infrared spectroscopy to predict and measure cochlear implant outcomes: a scoping review,” Brain Sci. 11(11), 1439 (2021). 10.3390/brainsci11111439 34827438 PMC8615917

[30] Mushtaq F. et al., “The benefit of cross-modal reorganization on speech perception in pediatric cochlear implant recipients revealed using functional near-infrared spectroscopy,” Front. Hum. Neurosci. 14, 308 (2020). 10.3389/fnhum.2020.00308 32922273 PMC7457128

[31] Lucarini G. et al., “Imaging the developing brain with near-infrared spectroscopy in cochlear implanted children,” Imaging Neurosci. 3, IMAG.a (2025). 10.1162/IMAG.a.90 PMC12330856 40800835

[32] Friederici A. D., “Neurophysiological markers of early language acquisition: from syllables to sentences,” Trends Cogn. Sci. 9(10), 481–488 (2005). TCSCFK 1364-6613 10.1016/j.tics.2005.08.008 16139558

[r33] Gemignani J. et al., “Reproducibility of infant fNIRS studies: a meta-analytic approach,” Neurophotonics 10(2), 023518 (2023). 10.1117/1.NPh.10.2.023518 36908681 PMC9997722

[r34] Dehaene-Lambertz G. Dehaene S. Hertz-Pannier L., “Functional neuroimaging of speech perception in infants,” Science 298(5600), 2013–2015 (2002). SCIEAS 0036-8075 10.1126/science.1077066 12471265

[r35] Pena M. et al., “Sounds and silence: an optical topography study of language recognition at birth,” Proc. Natl. Amer. Sci. 100(20), 11702–11705 (2003). 10.1073/pnas.1934290100 PMC208821 14500906

[r36] Poeppel D., “The neuroanatomic and neurophysiological infrastructure for speech and language,” Curr. Opin. Neurobiol. 28, 142–149 (2014). COPUEN 0959-4388 10.1016/j.conb.2014.07.005 25064048 PMC4177440

[37] Skeide M. A. Friederici A. D., “The ontogeny of the cortical language network,” Nat. Rev. Neurosci. 17(5), 323–332 (2016). NRNAAN 1471-003X 10.1038/nrn.2016.23 27040907

[38] Sato H. et al., “Cerebral hemodynamics in newborn infants exposed to speech sounds: a whole-head optical topography study,” Hum. Brain Mapp. 33(9), 2092–2103 (2012). HBRME7 1065-9471 10.1002/hbm.21350 21714036 PMC6870359

[r39] May L. et al., “Language and the newborn brain: does prenatal language experience shape the neonate neural response to speech?” Front. Psychol. 2, 222 (2011).1664-1078 10.3389/fpsyg.2011.00222 21960980 PMC3177294

[r40] May L. et al., “The specificity of the neural response to speech at birth,” Dev. Sci. 21(3), e12564 (2018). 10.1111/desc.12564 28503845

[r41] Vannasing P. et al., “Distinct hemispheric specializations for native and non-native languages in one-day-old newborns identified by fNIRS,” Neuropsychologia 84, 63–69 (2016). NUPSA6 0028-3932 10.1016/j.neuropsychologia.2016.01.038 26851309

[42] Zhang F. Gervain J. Roeyers H., “Developmental changes in the brain response to speech during the first year of life: a near-infrared spectroscopy study of Dutch-learning infants,” Infant Behav. Dev. 67, 101724 (2022). IBDEDP 10.1016/j.infbeh.2022.101724 35640398

[43] Ramus F. et al., “Language discrimination by human newborns and by cotton-top tamarin monkeys,” Science 288(5464), 349–351 (2000). SCIEAS 0036-8075 10.1126/science.288.5464.349 10764650

[44] Nazzi T. Bertoncini J. Mehler J., “Language discrimination by newborns: toward an understanding of the role of rhythm,” J. Exp. Psychol. Hum. Percept. Perform. 24(3), 756–766 (1998). JPHPDH 0096-1523 10.1037/0096-1523.24.3.756 9627414

[45] Werker J. F. Hensch T. K., “Critical periods in speech perception: new directions,” Annu. Rev. Psychol. 66, 173–196 (2015). ARPSAC 0066-4308 10.1146/annurev-psych-010814-015104 25251488

[46] Reh R. K. et al., “Critical period regulation across multiple timescales,” Proc. Natl. Acad. Sci. 117(38), 23242–23251 (2020). 10.1073/pnas.1820836117 32503914 PMC7519216

[47] Werker J. F., “Phonetic perceptual reorganization across the first year of life: looking back,” Infant Behav. Dev. 75, 101935 (2024). IBDEDP 10.1016/j.infbeh.2024.101935 38569416

[48] Choi J. Cutler A. Broersma M., “Early development of abstract language knowledge: evidence from perception–production transfer of birth-language memory,” R. Soc. Open Sci. 4(1), 160660 (2017). 10.1098/rsos.160660 28280567 PMC5319333

[49] Byers-Heinlein K. et al., “Effects of language mixing on bilingual children’s word learning,” Bilingualism: Lang. Cogn. 25(1), 55–69 (2022). LCPRET 10.1017/S1366728921000699 PMC8992731 35399292

[r50] Byers-Heinlein K. et al., “A multilab study of bilingual infants: exploring the preference for infant-directed speech,” Adv. Methods Pract. Psychol. Sci. 4(1), 2515245920974622 (2021). 10.1177/2515245920974622 PMC9273003 35821764

[51] Bosch L. Sebastian-Galles N., “Native-language recognition abilities in 4-month-old infants from monolingual and bilingual environments,” Cognition 65(1), 33–69 (1997). CGTNAU 0010-0277 10.1016/S0010-0277(97)00040-1 9455170

[52] Boersma P. Weenink D., “PRAAT, a system for doing phonetics by computer,” Glot Int. 5, 341–345 (2001).

[53] Nallet C. L. C., The Special Status of Language: Neural Processing of Speech in Newborns and Young Infants with Typical and Atypical Auditory Experience, Università degli studi di Padova (2024).

[54] Lloyd-Fox S. et al., “Coregistering functional near-infrared spectroscopy with underlying cortical areas in infants,” Neurophotonics 1(2), 025006 (2014). 10.1117/1.NPh.1.2.025006 25558463 PMC4280679

[r55] Shi F. et al., “Infant brain atlases from neonates to 1-and 2-year-olds,” PLoS One 6(4), e18746 (2011). POLNCL 1932-6203 10.1371/journal.pone.0018746 21533194 PMC3077403

[r56] Abboub N. Nazzi T. Gervain J., “Prosodic grouping at birth,” Brain Lang. 162, 46–59 (2016). 10.1016/j.bandl.2016.08.002 27567401

[57] Martinez‐Alvarez A. et al., “Newborns discriminate utterance‐level prosodic contours,” Dev. Sci. 26(2), e13304 (2023). 10.1111/desc.13304 35841609

[58] Gemignani J. Gervain J., “Comparing different pre-processing routines for infant fNIRS data,” Dev. Cogn. Neurosci. 48, 100943 (2021). 10.1016/j.dcn.2021.100943 33735718 PMC7985709

[59] Berent I. et al., “Infants differentially extract rules from language,” Sci. Rep. 11(1), 1–10 (2021). SRCEC3 2045-2322 10.1038/s41598-021-99539-8 34625613 PMC8501030

[60] Aguirre G. K. Zarahn E. D’Esposito M., “The variability of human, bold hemodynamic responses,” NeuroImage 8(4), 360–369 (1998). NEIMEF 1053-8119 10.1006/nimg.1998.0369 9811554

[61] Ashburner J. et al., SPM12 Manual, Vol. 2464, p. 53, Wellcome Trust Centre for Neuroimaging, London, UK (2014).

[62] Lloyd-Fox S. Blasi A. Elwell C. E., “Illuminating the developing brain: the past, present and future of functional near infrared spectroscopy,” Neurosci. Biobehav. Rev. 34, 269–284 (2009). NBREDE 0149-7634 10.1016/j.neubiorev.2009.07.008 19632270

[63] Minagawa-Kawai Y. et al., “Optical brain imaging reveals general auditory and language-specific processing in early infant development,” Cereb. Cortex 21(2), 254–261 (2010). 53OPAV 1047-3211 10.1093/cercor/bhq082 20497946 PMC3020578

[64] Gervain J. et al., “Near-infrared spectroscopy: a report from the McDonnell infant methodology consortium,” Dev. Cogn. Neurosci. 1(1), 22–46 (2011). 10.1016/j.dcn.2010.07.004 22436417 PMC6987576

[r65] Ayaz H. et al., “Optical imaging and spectroscopy for the study of the human brain: status report,” Neurophotonics 9(S2), S24001 (2022). 10.1117/1.NPh.9.S2.S24001 36052058 PMC9424749

[66] Minagawa-Kawai Y. et al., “Optical imaging of infants’ neurocognitive development: recent advances and perspectives,” Dev. Neurobiol. 68(6), 712–728 (2008). 10.1002/dneu.20618 18383545

[67] Bartha-Doering L. et al., “Absence of neural speech discrimination in preterm infants at term-equivalent age,” Dev. Cogn. Neurosci. 39, 100679 (2019). 10.1016/j.dcn.2019.100679 31437736 PMC6969359

[r68] Gervain J. et al., “The neonate brain detects speech structure,” Proc. Natl. Acad. Sci. U.S.A. 105(37), 14222–14227 (2008). 18768785 10.1073/pnas.0806530105 PMC2544605

[r69] Minagawa-Kawai Y. et al., “Assessing signal-driven mechanisms in neonates: brain responses to temporally and spectrally different sounds,” Front. Psychol. 2, 135 (2011).1664-1078 10.3389/fpsyg.2011.00135 21720538 PMC3118480

[70] Sato Y. Sogabe Y. Mazuka R., “Development of hemispheric specialization for lexical pitch-accent in Japanese infants,” J. Cogn. Neurosci. 22(11), 2503–2513 (2010). JCONEO 0898-929X 10.1162/jocn.2009.21377 19925204

